# Mechanosensitive Piezo1 is crucial for periosteal stem cell-mediated fracture healing

**DOI:** 10.7150/ijbs.71390

**Published:** 2022-06-13

**Authors:** Yunlu Liu, Hongtao Tian, Yuxiang Hu, Yulin Cao, Hui Song, Shenghui Lan, Zhipeng Dai, Wei Chen, Yingze Zhang, Zengwu Shao, Yong Liu, Wei Tong

**Affiliations:** 1 Department of Orthopedics, Union Hospital, Tongji Medical College, Huazhong University of Science and Technology, Wuhan 430022, Hubei, China.; 2 Department of Orthopedics, The Eighth People's Hospital, Jiangsu University, Shanghai 200235, China.; 3 Department of Orthopedics, Xuhui Branch of The Sixth People's Hospital, Shanghai Jiao Tong University, Shanghai 200233, China.; 4 Department of Orthopedics, Henan Provincial People's Hospital, Zhengzhou University, Zhengzhou 450003, Henan, China.; 5 Department of Orthopedics, The Third Hospital of Hebei Medical University, Shi Jiazhuang 050051, Hebei, China.; 6 NHC Key Laboratory of Intelligent Orthopedic Equipment (The Third Hospital of Hebei Medical University), Shi Jiazhuang 050051, Hebei, China.

**Keywords:** Piezo1, Mechanosensor, PSCs, Fracture healing, YAP

## Abstract

The biomechanical environment plays a dominant role in fracture healing, and Piezo1 is regarded as a major mechanosensor in bone homeostasis. However, the role of Piezo1 in fracture healing is not yet well characterized. In this study, we first delineated that Piezo1 is highly expressed in periosteal stem cells (PSCs) and their derived osteoblastic lineage cells and chondrocytes. Furthermore, downregulation of Piezo1 in callus leads to impaired fracture healing, while activation by its specific agonist promotes fracture healing through stimulation of PSC-modulated chondrogenesis and osteogenesis, along with accelerated cartilage-to-bone transformation. Interestingly, vascular endothelial growth factor A is upregulated after Yoda1 treatment of PSCs, indicating an indirect role of Piezo1 in angiogenesis. Mechanistically, activation of Piezo1 promotes expression of Yes-associated protein (YAP) and its nuclear localization in PSCs, which in turn increases the expression and nuclear localization of β-catenin. In detail, YAP directly interacts with β-catenin in the nucleus and forms a transcriptional YAP/β-catenin complex, which upregulates osteogenic, chondrogenic and angiogenic factors. Lastly, Yoda1 treatment significantly improves fracture healing in a delayed union mouse model generated by tail suspension. These findings indicate that Piezo1 is a potential therapeutic target for fracture delayed union or nonunion.

## Introduction

Bone fractures are one of the most common injuries to humans 1. Approximately 8 million people break a bone every year in the US alone 2. Despite advances in surgical treatment, approximately 5-10% of individuals will progress to delayed healing or nonunion 1, 2. However, no pharmacologic therapy is currently available for the treatment of fractures or to promote efficient bone healing. Thus, identification of new strategies to effectively promote bone healing so as to shorten the healing time is highly warranted.

There are three main phases of normal fracture healing: hematoma formation via inflammatory reaction, cartilage callus formation and finally cartilage remodeling to bone 1. Periosteal stem cells (PSCs) residing in the periosteum of the cortical bone, play critical roles in the whole healing process 3: In the early phase of healing, those cells are activated and migrate to the fracture line, where they undergo differentiation to form cartilage and new bone; they also release osteogenic and pro-angiogenic factors to regulate osteogenesis and angiogenesis 4, 5. Additionally, fracture healing is tightly regulated by the biomechanical environment 6, 7. Low to moderate interfragmentary strain promotes PSC-mediated callus formation 8, 9, whereas excessive or insufficient strain leads to abnormal cartilage callus and insufficient cartilage-to-bone transformation, and finally to delayed union or nonunion 6, 10. Therefore, a suitable biomechanical environment is essential for a normal healing process. However, the mechanism via which PSCs convert mechanical signals into biological signals and thus modulate fracture healing remains elusive.

Recent advances in mechanotransduction research have opened new avenues to understanding how cells respond to the mechanical properties of the microenvironment 11-13. Piezo, Piezo1 and Piezo2, proteins have been proposed as Ca^2+^-permeable cation channels that serve in mechanosensory transduction and play critical roles in diverse biological processes 14, 15. Piezo1 is mainly expressed in non-sensory tissues and mediates mechanical responses of various cell types, including endothelial cells 16, astrocytes 17, smooth muscle cells 18, pancreatic acinar cells 19, and red blood cells 20. Meanwhile, recent studies have shown that Piezo1 is a key determinant of bone homeostasis and metabolism. For instance, Piezo1 has been found to be required for mechanosensation and bone formation by osteoblastic lineage cells including bone marrow mesenchymal stem cells (BMSCs) 21-24. However, PSCs contributing to osteoblastic lineages cells and chondrocytes in the callus differ greatly from BMSCs 25, 26, and whether and how Piezo1 regulates their function in the fracture healing process remains uncharacterized.

In the current study, we first analyzed the cell clusters of the fracture callus by single-cell sequencing (scRNA-seq) and found that PSCs and their derived chondrocytes and osteoblastic lineage cells share the greatest percentage of bone-forming cells. Moreover, Piezo1 is highly expressed in PSCs and its osteoblastic lineage cells and chondrocytes. Next, downregulation of Piezo1 resulted in an impaired healing process, while administration of Yoda1, a Piezo1 agonist, accelerated fracture healing by promoting chondrogenesis and osteogenesis of PSCs through activation of the YAP-β-catenin signaling pathway. Finally, Yoda1 was found to mimic physical force and thus improve delayed fracture healing caused by mechanical unloading. Collectively, these observations revealed a new mechanism underlying how PSCs convert a mechanical signal to a biological signal and modulate the fracture-healing process, and thus suggest that activation of Piezo1 may be a potential therapeutic target for accelerating fracture healing.

## Materials and Methods

### Mice

Male C57BL/6J mice at 8 weeks old were purchased from Beijing SPF Biotechnology Co., Ltd. Rosa-tdTomato mice were purchased from Jiangsu GemPharmatech Co., Ltd., (Nanjing, China) and Gli1-CreER Rosa-tdTomato (Gli1/Tomato) mice were generated by breeding Rosa-tdTomato mice with Gli1-CreER mice obtained from Jackson Laboratory (Bar Harbor, ME, USA). All mice were bred and maintained under specific pathogen free conditions. In order to induce CreER activity in mice, tamoxifen (75 mg/kg/d) was injected subcutaneously every day for 5 days starting 5 days before the bone fracture. For fracture healing evaluation, mice in the treatment group were injected intraperitoneally with 5 mmol/kg Yoda1 (SML1558; Sigma, St. Louis, MO, 40 mM dissolved in DMSO then diluted with 5% ethanol) daily for five consecutive days per week until time of sacrifice (Figure 3A). The control mice received an equivalent volume of vehicle.

The animal research protocols were approved by the Animal Care and Ethics Committee at Huazhong University of Science and Technology (No. S2562).

### Human tissue specimens

Fresh callus tissue and paired bone tissue were obtained from five patients who underwent surgery for internal fixation of fractures at the Union Hospital, Tongji Medical College (Wuhan, China). The information of the samples is shown in Table S1. All human patient samples were obtained with consent under approval and oversight by the Ethics Committee of Tongji Medical College, Huazhong University of Science and Technology (No. S200).

### Femoral fracture model

The surgery was performed as previously described 27. Briefly, mice were anesthetized with pentobarbital sodium (50 mg/kg). Next, the right femur was sterilized and then a transverse fracture was created at the mid-shaft, and the fracture was fixed by inserting a sterilized 23-gauge needle into the medullary cavity. Fractured bones were harvested at different time points for micro-computed tomography (μCT), histology, and three-point bending assays (Figure 3A).

### scRNA-seq and analysis

At 10 days (n = 5) and 20 (n = 5) days post fracture (dpf), the callus was isolated from each mouse and dissociated into a single-cell suspension by enzyme digestion as described previously 28. Briefly, cDNA amplification and chromium library construction were carried out using the scRNA-seq library kit v3 (10 × Genomics, Pleasanton, CA, USA). Libraries were sequenced using an Illumina NovaSeq 6,000 (Illumina Inc., San Diego, CA, USA) with read length of 150 bp by the Wuhan Biobank Co., Ltd. The R package Seurat (Version 2.3.1) was used for data analysis 29. Briefly, single cells were filtered (nFeature 6,000, nCount 10,000, percent_mt 0.25) for downstream analysis. The cells were grouped and visualized using the uniform manifold approximation and projection (UMAP). Differentially expressed genes (DEGs) were defined with the threshold of adjusted *p*-value set at 0.05. Pseudotime and RNA velocity analysis were performed as previously reported 30.

### Intra-callus injection of lentivirus

The recombinant lentivirus to carry the interference vector of Piezo1 (Lv-shPiezo1-GFP) and control vector (Lv-NC-GFP) were obtained from GeneChem (Shanghai, China). In brief, lentivirus preparations were injected into multiple sites of the callus region at 4 days postoperation using a 15 μL syringe. On 20 dpf, the fractured femora samples were harvested for further study.

### µCT analysis

Femora were harvested at 7, 14, 21 and 28 dpf, and fixed in 4% paraformaldehyde (PFA). μCT analyses were performed using a high-resolution μCT scanner (Bruker SkyScan 1176, Karlsruhe, Germany). The 3-D reconstruction of the fracture callus was performed using CT-Vox 2.1. Three-dimensional morphological parameters including callus total volume (TV), callus bone volume (BV), BV/TV, and bone mineral density (BMD) were recorded and calculated using CTAN 1.12 software (Bruker).

### Mechanical testing

The fractured femora were harvested and the attached soft tissue was carefully removed. Next, a three-point bending test (span length, 10 mm; loading tip radius 0.3 mm; loading speed 1.8 mm/min) at the mid femur was preformed using an Instron 5542 (Instron, Norwood, MA, USA), and the peak loads were calculated from the load-to-failure curve.

### Histology and immunohistochemistry (IHC)

Mouse femur samples were harvested, fixed in 4% PFA, and embedded in paraffin after decalcification with 10% EDTA for 21 days. To obtain frozen sections, the samples were fixed with 4% PFA, soaked in 30% sucrose and embedded in OCT compound. A series of 10 μm sections were subjected to IHC, as well as Safranin-O/Fast green, and hematoxylin and eosin staining. For IHC staining, antigen retrieval was performed, then the sections were incubated with primary antibodies, including anti-Ki67 (1:200, 14-5698-95, Thermo Fisher Scientific, Waltham, MA, USA), anti-Osteocalcin (1:100, PB1008, Boster, Wuhan, China), anti-β-catenin (1:100, 610154, BD Biosciences, Franklin Lakes, NJ, USA), anti-Piezo1 (1:100, 15939-1-AP, Proteintech, Rosemont, IL, USA), anti-Piezo2 (1:100, 26205-1-AP, Proteintech), anti-YAP (1:100, 14074, Cell Signaling Technology, Danvers, MA, USA), anti-Endomucin (1:100, sc-65495, Santa Cruz Biotechnology, Santa Cruz, CA, USA), anti-CD31 (1:50, 563607, BD Biosciences) and anti-Col2α1 (1:100, bs-11929R, Bioss Antibodies, Woburn, MA, USA). After rinsing twice, the sections were incubated with the appropriate secondary antibodies. For immunofluorescent imaging, the sections were counterstained with 4',6-diamidino-2-phenylindole (DAPI; Sigma-Aldrich, St Louis, MO, USA) and imaged using confocal microscopy (Nikon A1; Nikon, Tokyo, Japan).

### Cell isolation, culture and treatment

PSCs were isolated from intact femora and tibiae from 8-week-old mice as described previously 31. Briefly, the adherent soft tissue and both ends of each bone were carefully removed from the bone. The remaining bone fragments were digested enzymatically (0.2% collagenase A, 0.25% trypsin in PBS) at 37 °C in an orbital shaker for 1 h. The culture medium was alpha-minimum essential medium (αMEM; Gibco, Grand Island, NY, USA) enriched with 15% fetal bovine serum (FBS; Gibco), 55 mM β-mercaptoethanol, 2 mM glutamine, and 1% penicillin/streptomycin (Gibco). All experiments were performed using passage 1 (P1) PSCs.

Yoda1 was resuspended in 50 mM DMSO and diluted in culture medium to the final concentration. To study the effect of Yoda1 on differentiation, Yoda1 (0.3 μM) was added to cell culture medium after two days of induction. To test the effects of YAP inhibitors (verteporfin; S1786, Selleck Chemicals, Houston, TX, USA) on migration and differentiation, and the effects of β-catenin inhibitors (LF3; S8474, Selleck Chemicals), the cells were pretreated with the indicated concentrations of inhibitors for 6 h and treatment then continued throughout the whole assay.

For osteogenic differentiation, PSCs were seeded at a density of 3.0 × 10^4^/well and cultured in osteogenic-inducing medium (αMEM containing 10% FBS, 10 nM dexamethasone, 50 mg/mL ascorbic acid, 1% penicillin/streptomycin and 10 mM β-glycerophosphate) that was changed every 3 days. Calcium deposition was detected by Alizarin Red S staining after 14 days of induction.

For chondrogenic differentiation, PSCs were centrifuged into pellets (2.0 × 10^4^ cells/pellet) in polypropylene tubes and cultured in chondrogenic medium (high-glucose DMEM containing 40 mg/mL L-proline, 0.1 mM dexamethasone, 1% penicillin/streptomycin, 100 mg/mL sodium pyruvate, 50 mg/mL ascorbate-2-phosphate, 1% ITSp Premix). Before chondrogenic induction, 10 ng/mL TGF-β1 (PeproTech, Rocky Hill, NJ, USA) was freshly added to differentiation medium, which was changed every 3 days. The cell pellets were cultured for 21 days and then fixed in 4% PFA and then embedded in paraffin. To evaluate collagen deposition, paraffin sections were stained with alcian blue.

### Cell lines

Human umbilical vein endothelial cells (HUVECs) were cultured as previously described 32 in endothelial cell medium containing 10% FBS at 37 °C with 5% CO_2_.

### Cell transfection

The cells were seeded into 6-well plates (2 × 10^4^ cells/well) and then transfected with lentivirus (Lv)-shPiezo1 (Genechem Company, Shanghai, China) at a multiplicity of infection (MOI) of 50 for 12 h. The Lv-NC served as negative control. After 36 h of infection, cells were subjected to puromycin (2.5 µg/mL) selection for 7 days. Before further experiments, the efficacy of the gene knockdown was measured by RT-PCR and western blot analysis.

### Measurement of calcium concentration

The cells were incubated with 1 µM fluo-4 AM (Beyotime biotechnology, Jiangsu, China) for 1 h at 37 °C. After rinsing twice, the cells were incubated for another 20 minutes at 37 °C. Then, the mean fluorescence intensity (MFI) before and after Yoda1 stimulation were recorded by flow cytometry (BD Biosciences) and analyzed using FlowJo 10.6 software (Beckman Coulter Life Sciences, Indianapolis, IN, USA).

### Cell counting kit-8 (CCK-8) assay

To document cell viability after Yoda1 treatment, the CCK-8 colorimetric assay (Dojindo, Japan) was applied as described previously 33, 34. Briefly, cells (5 × 10^3^ cells/well) were resuspended and seeded into 96-well plates. After incubation for 48 h, cells were exposed to Yoda1 as described above. Afterwards, the supernatants were removed and replaced with 100 μL of fresh medium containing 10 μL of CCK-8 solution. After incubation for 2 h at 37 °C in the dark, the absorbance was measured at 450 nm using a microplate reader (Biotek, Winooski, VT, USA).

### Conditioned medium

PSCs (1 × 10^6^) were cultured in 6-well plates and exposed to Yoda1 for 24 h, and then the culture medium was changed to fresh medium with or without FBS. After 24 h, the conditioned medium was collected and used for migration and tube formation assays of HUVECs.

### Migration assay

The cells (2 × 10^4^ cells/well) were seeded into the upper chamber of a Transwell® plate (Corning Costar, Rochester, NY) with serum-free medium. Fresh conditioned medium containing 20% FBS was added into the lower chamber of each well and incubated for 24 h at 37 °C with 5% CO_2_. The cells were then fixed with 4% PFA and stained with 0.5% crystal violet (Sigma-Aldrich) for 20 min, then imaged using a microscope (Olympus IX71, Tokyo, Japan).

### Tube formation assay

The tube formation assay was performed as previously described 35. Briefly, 96-well plates were coated with 50 μL Matrigel (BD Biosciences) and allowed to solidify at 37 °C for 1 h. Afterwards, 2 × 10^4^ HUVECs were seeded into each well, and incubated at 37 °C with 5% CO_2_ for 4 h. The cells were stained with calcein acetoxymethyl ester (Calcein AM, 2 μM, Sigma-Aldrich) and photographed by fluorescence microscopy. The corresponding areas and number of tubes formed were analyzed using ImageJ software (RRID: SCR_003070).

### Real-time polymerase chain reaction (RT-PCR)

Total RNA was prepared from cells or tissues using Trizol reagent (TaKaRa Bio, Tokyo, Japan) and reverse transcribed to cDNA using a reverse-transcription kit (TaKaRa Bio). The expression levels of mRNA were analyzed by RT-PCR using TB Green Premix Ex Taq II (TaKaRa Bio). Gene-specific primer sequences for RT-PCR are provided in Table S2.

### RNA sequencing

Total RNA was isolated from callus samples at 10 dpf using Trizol (TaKaRa Bio) and evaluated using an Agilent 2100 BioAnalyzer (Agilent Technologies, Santa Clara, CA, USA). RNA sequencing libraries were generated and sequenced by CapitalBio Technology (Beijing, China). Statistical analysis of DEGs was performed using the DESeq2 package, and the threshold of adjusted *p*-value was 0.01. Heatmaps of DEGs were further clustered by hierarchical clustering and then visualized by TreeView software and analyzed using the DAVID Bioinformatics Resources 6.8.

### Immunofluorescence staining

Cells cultured on slides were fixed with 4% PFA and blocked with 5% bovine serum albumin (BSA). The primary antibody was diluted in 5% BSA and incubated overnight at 4 °C. After rinsing, the cells were incubated with a fluorophore-conjugated secondary antibody for 1 h and counterstained with DAPI in the dark for 5 min. Images were acquired on a laser-scanning confocal microscope (Nikon A1).

### Western blot analysis and co-immunoprecipitation (Co-IP)

Details of the method for western blot analysis have been previously described 33, 34. Antibodies against Piezo1 (1:1,000, 15939-1-AP, Proteintech), YAP (1:1,000, 14074, Cell Signaling Technology), β-catenin (1:1,000, 610154, BD Biosciences), BMP2 (1:1,000, AF5163, Affinity Biosciences), VEGFA (1:1,000, ab214424, Abcam), H3 (1:1,000, ab32356, Abcam) and β-actin (1:2,000, 66009-1-Ig, Proteintech) were used as primary antibodies.

For immunoprecipitation, the nuclear and cytoplasmic lysates were precipitated with the indicated antibody or control IgG for 2 h. The immune complexes were collected and analyzed by western blotting according to standard procedures.

### Verteporfin administration

Verteporfin (S1786; Selleck Chemicals) was dissolved in DMSO (100 mg/mL) then freshly diluted with PBS before use and was given at 24 h after fracture modeling. Briefly, the mice in the YAP inhibitor group were intraperitoneally injected with verteporfin (100 mg/kg) every other day. Control mice were treated with an equal volume of vehicle. Bone tissues were harvested at 2 weeks after treatment and fixed with 4% PFA for further analysis.

### Tail suspension model

Tail suspension experiments were performed as previously described 21. Briefly, the mice were suspended in separate cages with their heads tilted down at an angle of about 30 degrees. The suspension angle was adjusted to prevent the hind limbs from contacting the cage floor. The fractured femora were harvested, and the needle was removed at 10 or 20 dpf. μCT, bone sectioning, and staining were further performed on these bone samples.

### Statistical analysis

Data analysis was performed using GraphPad Prism 8 software (GraphPad Software Inc., La Jolla, CA). Data are presented as mean values ± standard deviation (SD) of at least three independent experiments. For comparisons between two groups the two-tailed Student's t test was used. For multiple comparisons between more than two groups, data were analyzed by one-way analysis of variance (ANOVA) with the Bonferroni post hoc test. *P* < 0.05 was considered statistically significant. Statistically significant differences are indicated as follows: * for *p* < 0.05 and ** for *p* < 0.01. *** for *p* < 0.001.

## Results

### The expression pattern of Piezo1 in fracture healing

**Figure 1:**
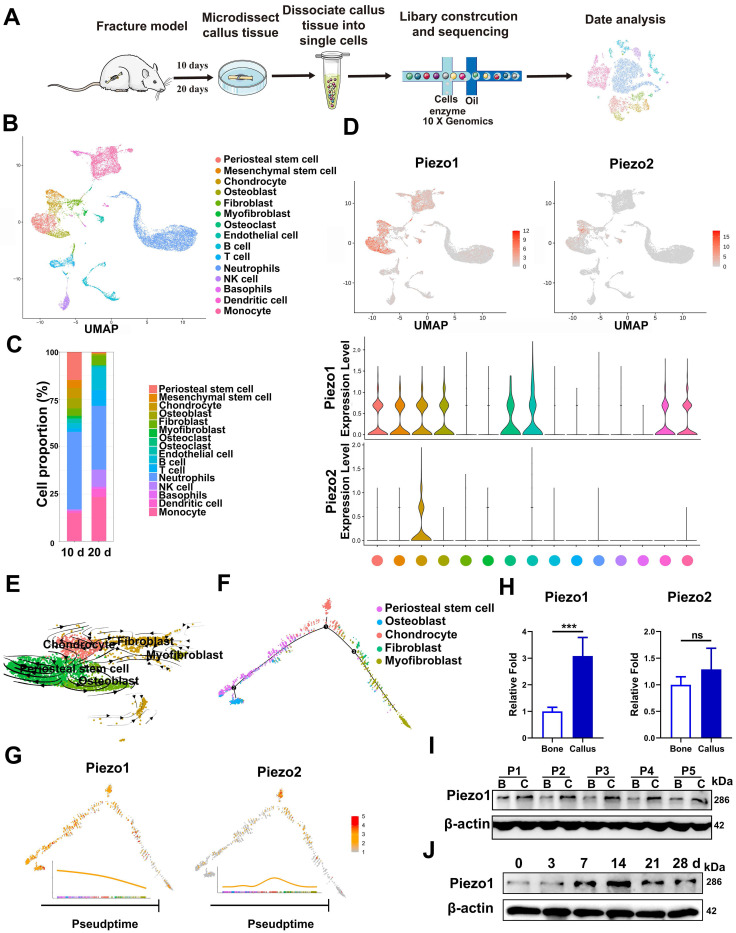
** Expression pattern of Piezo1 in fracture healing. (A)** Schematic diagram of cell isolation, cell processing, capture by droplet-based device, sequencing, and downstream analysis. **(B)** UMAP plot revealed cellular heterogeneity with 15 distinct clusters of cells identified and color-coded. The general identity of each cell cluster is defined on the right. **(C)** Stacked bar chart showing the percentages of each cell type within callus tissue quantified at 10 and 20 days post-fracture based on UMAP distribution. **(D)** Feature plots (upper image) and violin plots (lower image) showing the expression distribution of Piezo1 and Piezo2 genes. Expression levels for each cell are color-coded and overlaid onto the UMAP plot. **(E)** Projection of periosteal stem cells, osteoblasts, chondrocytes, fibroblasts and myofibroblasts using RNA velocity analysis with the time derivatives of the gene expression state. **(F)** Pseudotime ordering of periosteal stem cells, osteoblasts, chondrocytes, fibroblasts and myofibroblasts, arranged into a major trajectory. **(G)** Feature plots showing the expression distribution of Piezo1 and Piezo2 across pseudotime. Expression levels for each cell are color-coded with the highest expression level colored red. **(H)** RT-PCR analysis of Piezo1 and Piezo2 mRNA expression levels in callus and corresponding bone tissues. **p < 0.01. Data are mean ± SD, n = 5 per group. **(I)** Western blot analysis of Piezo1 protein expression level in human callus and corresponding bone tissues. B, bone tissue; C, callus tissue. **(J)** Western blot analysis of Piezo1 protein expression level in fracture callus at 0, 3, 7, 14, 21 and 28 days post-fracture in mice, Data are mean ± SD, n = 3 per group.

We first characterized the change of cell clusters during fracture healing by scRNA-seq, which was performed on unsorted cells from bone callus at 10 and 20 dpf (Figure 1A), as those time-points coincide with the callus formation and remodeling stages 1, 36. After stringent cell filtration, the high-quality transcriptomes of 22,147 single cells collected from bone callus at 10 (n = 5) and 20 (n = 5) dpf were retained for subsequent analyses. A total of 15 cell clusters were identified in our database (Figure 1B). The cell types were annotated according to the expression of marker genes (Figure S1A). As expected, scRNAseq of the total fracture callus identified the major immune cell clusters, including neutrophils, basophils, monocytes, dendritic cells, T cells, NK cells and B cells. Consistent with previous reports 37-39, we identified two stem cell populations, including periosteal stem cells (Cd200, AlphaV, Ctsk) and mesenchymal stem cells (Ly6a, Thy1), followed by chondrocytes (Acan, Sox9), osteoblasts (Runx2, Ibsp), fibroblasts (Col1α1, Col3α1 and Col5α1), putative myofibroblasts (Acta2, Mly9), osteoclasts (Ctsk, Mmp9) and endothelial cells (Cdh5, Pecam). Additionally, bone-forming cells including PSCs (14.7%), chondrocytes (5.4%) and osteoblasts (5.4%) were found to be more abundant in 10 dpf bone callus compared with 20 dpf callus. Interestingly, the NK cells, T cells and B cells were abundant in 20 dpf callus in the scRNA-seq datasets (Figure 1C). In summary, the data set covers almost all known cell types involved in fracture repair 38, and allowed us to explore the composition of cells at different stages. Notably, Piezo1 was highly expressed in PSCs, MSCs, chondrocytes, osteoblasts, fibroblasts, myofibroblasts, osteoclasts and endothelial cells. In contrast, Piezo2 was expressed only in some chondrocytes (Figure 1D). Next, we performed pseudo-time analysis using RNA velocity analysis (Figure 1E) and Monocle2 (Figure 1F), and found that PSCs are the ancestor of osteoblasts, chondrocytes, fibroblasts and myofibroblasts in the dataset. Interestingly, cells expressing Piezo1 preferentially distributed at the beginning and middle of paths, while Piezo2 increased in expression levels at the middle of these paths (Figure 1G). These findings suggest that Piezo1 may play a significant role in the differentiation of PSCs.

In order to verify the changes of Piezo protein expression in callus tissue, we then collected fresh callus tissues and adjacent bone tissue samples from five patients who were undergoing internal fracture fixation surgery after fractures. The histories of the donors and the injury time are shown in Supplementary Table 1. RT-PCR confirmed that the expression levels of Piezo1 were higher in callus than in normal bone tissue. Meanwhile, Piezo2 mRNA expression levels in the fracture callus were not significantly altered (Figure 1H). According to IHC assay, the expression level of Piezo1 was higher than that of Piezo2 in callus samples compared with corresponding bone tissue (Figure S2A). Analysis of protein expression showed that Piezo1 was also higher in fracture callus than in the matched bone tissue (Figure 1I), illustrating the general relevance of a possible role of Piezo1 in fracture healing.

In order to understand the temporal expression pattern of Piezo1 during the healing process, we collected callus tissue from different periods using a mouse femoral fracture model. As shown in Figure 1J, Piezo1 expression increased after fracture, peaked by day 14 and decreased by days 21 and 28 in fractured bones. We then explored the expression pattern in fractured femora using IHC staining, and found that Piezo1-expressing positive cells were detected within all of the callus areas, including the cartilaginous callus and the intramembranous bone formation areas, while Piezo2-positive cells were only detected within the cartilaginous callus (Figure S2B and C). These results suggest that Piezo1 might play a dominant role in the fracture healing process.

### Downregulation of Piezo1 in callus resulted in delayed fracture healing in mice

**Figure 2:**
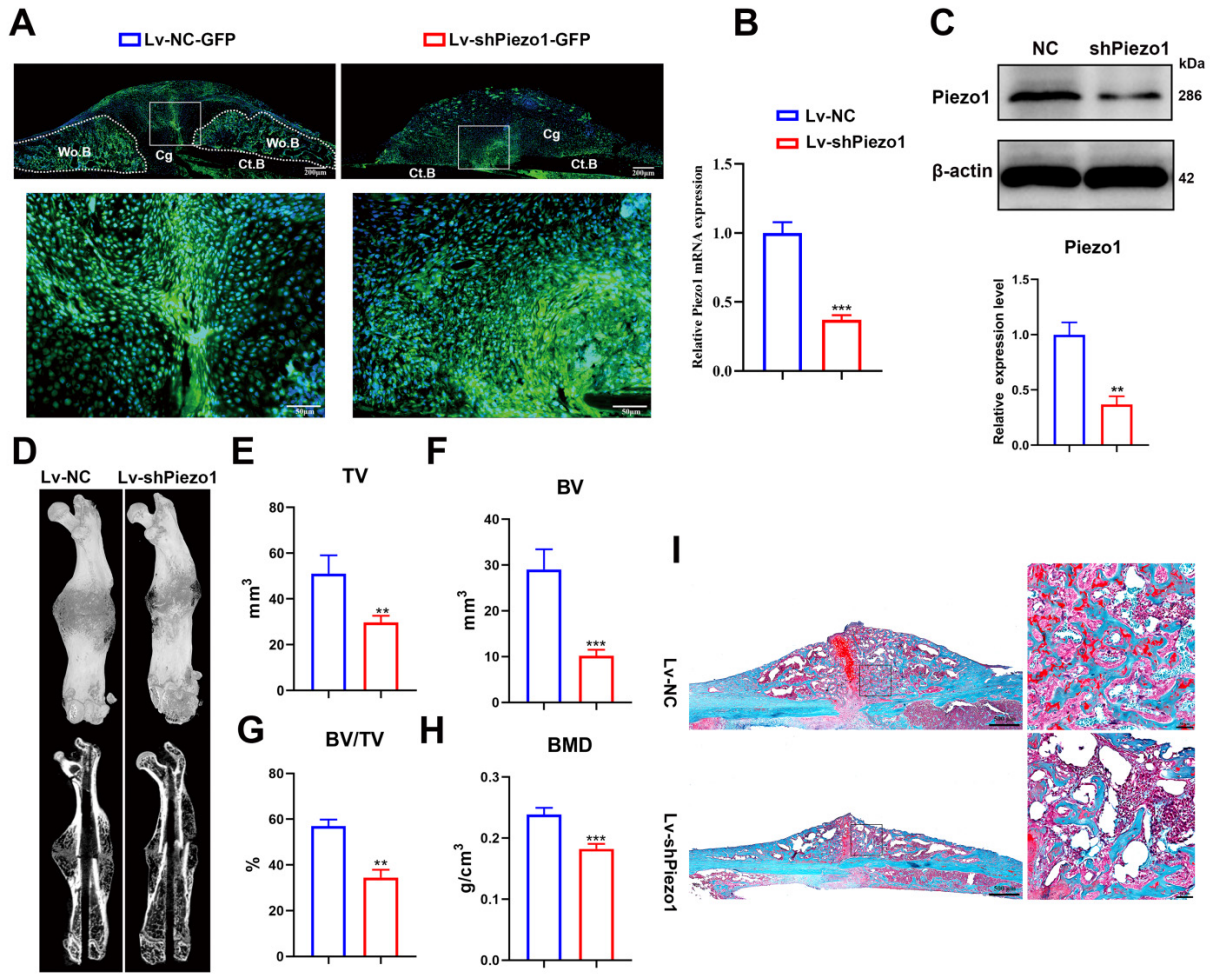
** Downregulation of Piezo1 in callus impaired bone healing. (A)** Image of Lv-transfected callus area, the fluorescent signal (green) represents Lv-NC-GFP and Lv-shPiezo1-GFP transduced cells at 10 days after intra-callus injection. The nuclei were stained with DAPI (blue). Scale bars = 200 µm (upper image); 50 µm (lower image). **(B)** RT-PCR analysis of knockdown efficiency of Lv-shPiezo1 in callus. Data are mean ± SD, n = 4 per group, ***p < 0.001. **(C)** Western blot analysis of Piezo1 expression in the Piezo1 shRNA group and the NC group at day 17 after injection. Data are mean ± SD, n = 3 per group, **p < 0.01. **(D)** Representative µCT three-dimensional reconstructions and coronal cross-sectional images of NC and Piezo1-shRNA-group mice at 21 days after fracture. μCT analysis of fracture calluses for** (E)** callus tissue volume (TV), **(F)** bone volume (BV), **(G)** bone volume fraction (BV/TV), and **(H)** bone mineral density (BMD) of fracture calluses at 21 days post-fracture. Data are mean ± SD, n = 4 per group, **p < 0.01, ***p < 0.001. **(I)** Representative Safranin O/Fast green staining images of fracture calluses at 21 days post-fracture. Scale bar = 500 µm (left); 50 µm (right). Ct.B = cortical bone; Cg = cartilage; Wo.B = woven bone.

To validate the role of Piezo1 in fracture healing, we transduced Lv-shPiezo1-GFP into callus tissue at 4 dpf. Green signals indicated that the Lv-NC-GFP or Lv-shPiezo1-GFP (Figure 2A) was successfully transduced into callus tissue at 10 days after injection. The expression of Piezo1 mRNA was significantly knocked down by almost 70% by Piezo1 shRNA when compared with NC shRNA at day 17 after injection (Figure 2B). The downregulation of Piezo1 protein was confirmed by western blot analysis (Figure 2C). Meanwhile 3D reconstructions of calluses at 21 dpf showed that, compared to the NC group, the Piezo1-knockdown group continued to develop a smaller and less mineralized callus with a persistent unmineralized fracture gap (Figure 2D). Quantitative analysis of μCT images showed that TV decreased significantly in the Piezo1-knockdown group at 21 dpf (Figure. 2E). Additionally, calluses in the Piezo1-knockdown group possessed 65% lower BV and 40% lower BV/TV than those in NC controls (Figure 2F and G). The BMD was also lower in the Piezo1-knockdown callus (Figure 2H). Safranin O/Fast green staining showed that both a reduced callus size and fewer bone trabeculae were formed in Piezo1-knockdown callus (Figure 2I). Taken together, these data showed that union was delayed, and callus mineralization was impaired when Piezo1 was downregulated, illustrating that Piezo1 is critical to bone formation during the healing cascade.

### Activation of Piezo1 accelerated fracture healing

**Figure 3:**
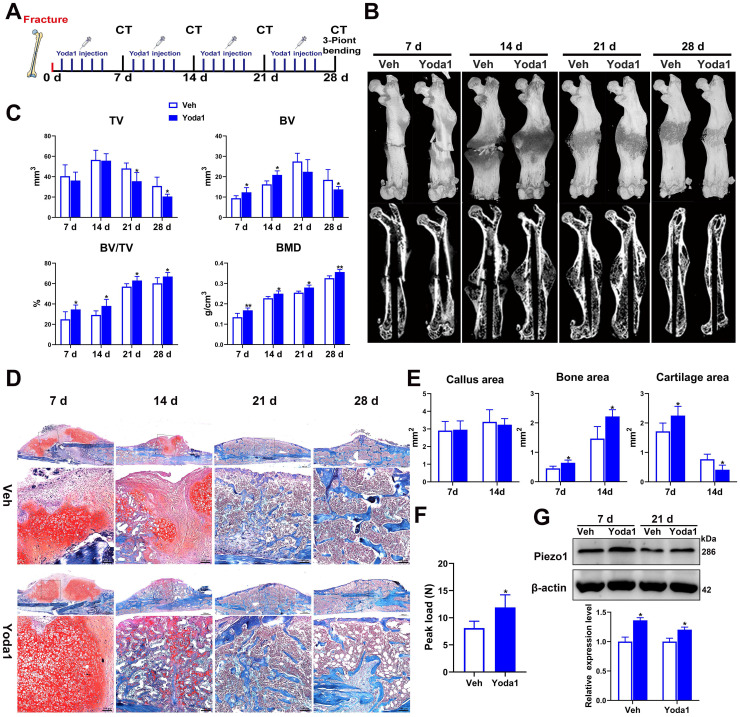
** Activation of Piezo1 accelerated fracture healing. (A)** Schematic design of animal experiments. **(B)** Representative three-dimensional reconstructions and coronal cross-sectional images of fracture calluses at 7, 14, 21, and 28 days post-fracture. **(C)** Callus tissue volume (TV), bone volume (BV), bone volume fraction (BV/TV) and bone mineral density (BMD) of fracture calluses at 7, 14, 21, and 28 days post-fracture. Data are mean ± SD, n = 6 per group, *p < 0.05, **p < 0.01. **(D)** Representative Safranin O/Fast green staining images of fracture calluses at 7, 14, 21, and 28 days post-fracture. Scale bar = 500 µm (upper image); 100 µm (lower image). **(E)** Callus area, cartilage area, and bone area measured at the fracture callus in vehicle- and Yoda1-treated mice at 7 and 14 days post-fracture. Data are mean ± SD, n = 4 per group, *p < 0.05. **(F)** Three-point bending test was performed on femora at 28 days post-fracture. Data are mean ± SD, n = 6 per group, *p < 0.05. **(G)** Western blot analysis of Piezo1 expression levels in fracture callus at 7 and 21 days post-fracture in mice. Data are mean ± SD, n = 3 per group, *p < 0.05. Veh = vehicle.

To further investigate the role of Piezo1 in fracture healing, the effect of a Piezo1 channel specific activator (Yoda1) 40 was determined using a mouse femoral fracture model (Figure 3A). According to the μCT analysis, the fracture gap was indistinct at 14 dpf and the fracture line was nearly invisible at 28 dpf in the Yoda1-treated group compared with the vehicle group, confirming the beneficial role of Piezo1 channel activation in the fracture-healing process (Figure 3B). Additionally, μCT analysis showed that there was no difference in tissue volume between the vehicle group and the Yoda1-treated group at 7 and 14 dpf, but at 21 dpf, TV decreased gradually in the treated group, indicating progressive callus remodeling (Figure 3C). Moreover, compared to the vehicle group, the BV, BV/TV and BMD were all significantly increased in the Yoda1-treated group at both early (7, 14 dpf) and late (21, 28 dpf) time points (Figure 3C), suggesting increased mineralization in bone repair.

Next, we performed histological analysis to investigate the cellular mechanisms involved in accelerating fracture repair. Accordingly, Yoda1-treated mice displayed promotion of the healing process from day 7 onwards, with accelerated formation of a cartilaginous callus adjacent to the fracture line, and prominent absorption of the cartilage callus and initiation of mineralization at 14 dpf, while the control mice displayed smaller areas of cartilage and bone in the callus (Figure 3D). Quantification of cartilage distribution indicated that the calluses of Yoda1-treated mice contained more cartilage than controls at 7 dpf. Surprisingly, cartilage almost disappeared by day 14, while it was still largely measurable in vehicle-treated mice. On the other hand, total osseous tissue (Figure 3E) showed increases of 42% and 51% of total osseous tissue after Yoda1 treatment on days 7 and 14 respectively, indicating that the bone formation process started to accelerate in the early healing phases. In the Yoda1-treated group, the fracture healing was basically completed at 28 dpf, showing the lamellar structure of cortical bone. At this time point, there was still massive woven bone around the fracture site in the vehicle group (Figure 3D).

We next tested the biomechanical properties of the fractured femur by three-point bending test. Importantly, the maximum bending loads of fractured femora were 1.5-fold higher than controls in Yoda1-treated mice at 28 dpf (Figure 3F). Interestingly, we found that Yoda1 treatment significantly increased the expression of Piezo1 in callus at 7 and 21 dpf (Figure 3G). Moreover, Yoda1 treatment did not alter the body weight, microscopic morphology of the main organs (heart, liver, spleen, lung and kidney), or the liver and kidney function of the mice (Figure S3A-C). All these results demonstrated that Yoda1 treatment significantly improved the fracture healing process, and this treatment was harmless* in vivo*.

### Yoda1 treatment promoted migration and differentiation of PSCs

**Figure 4:**
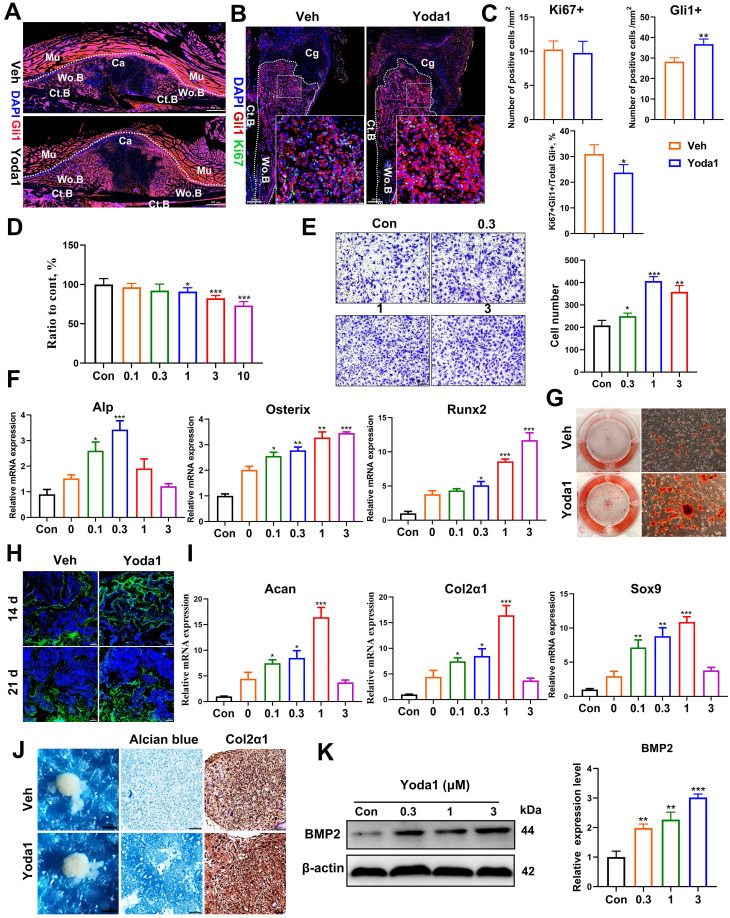
** Activation of Piezo1 enhanced bone regeneration and remodeling of the fracture callus. (A)** Fluorescence images of femoral sections from Gli1/Tomato mice (n = 3) at 10 days post-fracture. Scale bar = 500 µm. **(B)** Immunohistochemical analysis of fracture callus on day 10, including staining for Ki67 (green) and Gli1(red). Dotted lines outline the margins of woven bone in the fracture callus. Scale bar = 200 µm; 20 µm. **(C)** Comparison of the number of Gli1-postitive cells and Ki67-positive cells in the vehicle- and Yoda1-treated groups. Data are means ± SD, n = 4 per group, *p < 0.05, **p < 0.01. **(D)** PSCs were treated with Yoda1 for 24 h at a concentration ranging between 0.1 to 10 µM, and cell proliferation at each concentration was measured using the CCK-8 assay. Data are means ± SD of three independent experiments, *p < 0.05, ***p < 0.001. **(E)** Representative micrographs (left) and quantification (right) of the transwell migration assay in PSCs stimulated with Yoda1 for 24 hours. Scale bars = 100 µm. Data are means ± SD of three independent experiments, *p < 0.05, **p < 0.01, ***p < 0.001. **(F)** RT-PCR analysis of osteogenic marker gene expression in PSCs harvested after 10 days of culture in osteogenic medium with or without Yoda1 treatment. Data are means ± SD of three independent experiments, *p < 0.05, **p < 0.01, ***p < 0.001. **(G)** Alizarin red S staining of cells cultured in osteogenic medium for 2 weeks with or without Yoda1 (0.3 µM). **(H)** Representative immunofluorescent images of cells stained for osteocalcin (OCN) at 14 and 21 days post-fracture. Scale bar = 50 µm. **(I)** RT-PCR analysis of chondrogenic marker gene expression in PSCs harvested after 10 days of culture in chondrogenic medium with or without Yoda1 treatment. Data are means ± SD of three independent experiments, *p < 0.05, **p < 0.01, ***p < 0.001. **(J)** Images of cell pellets formed by 3D-cultured PSCs after culture in chondrogenic medium for 3 weeks with or without Yoda1 (0.3 µM) (1st column). Scale bar = 1 mm. Alcian blue staining of sections from chondrogenic cell pellets (2nd column). Scale bar = 50 µm. IHC staining of Col2α1 in sections of chondrogenic cell pellets (3rd column). Scale bar = 50 µm. **(K)** Western blotting analysis of BMP2 protein expression in PSCs. Cells were stimulated with different concentrations of Yoda1 or vehicle for 24 h, Data are means ± SD of three independent experiments, **p < 0.01, ***p < 0.001. Ct.B = cortical bone; Cg = cartilage; Ca = callus; Mu = muscle; Veh = vehicle; Wo.B = woven bone.

The majority of stem cells recruited to the fracture site are derived locally from the periosteum and the bone marrow 38. We and others revealed that PSCs labeled by Gli1 contributed greatly to osteoblast lineage cells during callus formation 31, 41, so we fate-mapped Gli1+ PSCs *in vivo* using Gli1-CreER; tdTomato mice during fracture healing. Gli1 Td+ cells were significantly increased in callus tissue in the Yoda1-treated group compared with the vehicle group (Figure 4A). To our surprise, there was no significant difference in the number of Ki67-positive cells (Figure 4B, C). This suggested that Yoda1 treatment accelerated the migration of PSCs to the fracture line but appeared to have no effect on cell proliferation (Figure 4C). To validate this, we isolated PSCs from 8-week-old mice and found that Piezo1 was highly expressed in PSCs, being especially expressed in the plasma membrane (Figure S4A), and treatment with Yoda1 resulted in rapid calcium influx in PSCs (Figure S4B). As shown in Figure 4D, the application of Yoda1, in part, reduced cell proliferation in a dose-dependent manner. To further demonstrate the function of Piezo1 in PSCs, we performed a transwell assay and found that Yoda1 significantly promoted the migration ability of PSCs in a dose-dependent manner (Figure 4E).

Next, we investigated whether Piezo1 activation affected PSC differentiation. As shown in Figure 4F, treating PSCs with Yoda1 increased osteogenic differentiation as shown by the expression levels of osteoblastic markers (Alp, Runx2 and Osterix). Furthermore, Alizarin Red S staining revealed that Yoda1 promoted nodule formation by PSCs (Figure 4G). To confirm our *in vitro* data, osteocalcin (OCN) immunofluorescence staining was performed on fractured callus at 14 and 21 dpf, and significantly more OCN-positive cells were found in Yoda1-treated callus compared to the vehicle-treated group (Figure 4H). Interestingly, the chondrogenic differentiation ability of PSCs was also enhanced by Yoda1, indicated by upregulated chondrogenic markers (Acan, Col2α1 and Sox9) (Figure 4I). However, incubation of PSCs with Yoda1 (0.3 μM) showed no effect on pellet size, but resulted in more GAG production, and significantly increased Col2α1 expression in the pellet (Figure 4J). Additionally, treatment of PSCs with Yoda1 for 24 h significantly induced bone morphogenetic protein 2 (BMP2) expression in a dose-dependent manner (Figure 4K). Thus, our results showed that Piezo1 activation accelerated the migration and differentiation of PSCs at the fracture region, thereby facilitating callus formation and consequent fracture repair.

### Piezo1 activation in PSCs indirectly promoted angiogenesis in fracture callus

**Figure 5:**
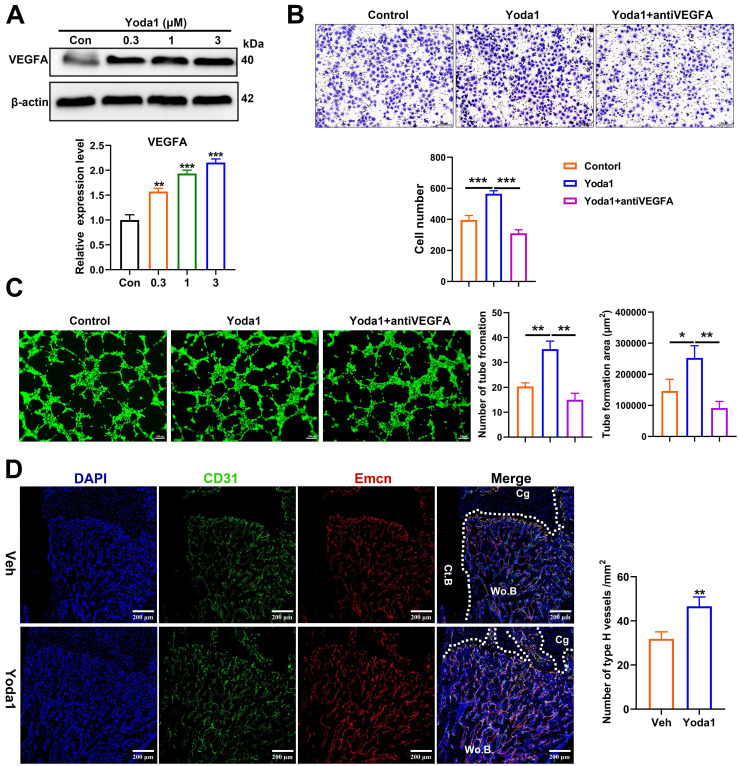
** Piezo1 activation in PSCs promoted angiogenesis in fracture callus. (A)** Western blotting analysis of VEGFA protein expression in PSCs. Cells were stimulated with different concentrations of Yoda1 or vehicle for 24 h, Data are means ± SD of three independent experiments, **p < 0.01, ***p < 0.001. **(B)** Representative micrographs (left) and quantification (right) of the transwell migration assay in HUVECs stimulated with conditioned medium collected from PSCs with or without Yoda1 treatment. Scale bars = 100 µm. Data are means ± SD of three independent experiments, ***p < 0.001. **(C)** Representative images of capillary-like tube formation (left) and quantification (right) of the tube formation number and area in HUVECs stimulated with conditioned medium collected from PSCs with or without Yoda1 treatment. Scale bars = 100 µm. Data are means ± SD of three independent experiments, *p < 0.05, **p < 0.01. **(D)** Representative CD31 (green), and Emcn (red) co-immunostaining images of fracture callus at 10 days post-fracture counterstained by DAPI (blue). Dotted lines outline the margins of woven bone in the fracture callus. Scale bars = 20 µm. Data are mean ± SD, n = 3 per group, **p < 0.01. Ct.B = cortical bone; Cg = cartilage; Veh = vehicle; Wo.B = woven bone.

Osteogenesis and angiogenesis are intimately coupled during bone regeneration 42. We next examined whether Piezo1 activation in PSCs affected angiogenesis. Notably, treatment of PSCs with Yoda1 for 24 h significantly induced vascular endothelial growth factor A (VEGFA) expression in a dose-dependent manner (Figure 5A). To validate the possible angiogenic function of Piezo1 activation in PSCs, conditioned medium from PSCs after Yoda1 treatment was collected. As shown in Figure 5B and C, the migration and tube formation of HUVECs was significantly enhanced by conditioned medium from Yoda1-treated PSCs compared to vehicle-treated cells, and this increase could be significantly blocked by VEGFA inhibition. In line with this, we found that the formation of type H vessels (CD31^hi^Emcn^hi^) in the fracture callus was significantly increased in the Yoda1-treated group (Figure 5D). These data demonstrated that Piezo1 activation in PSCs can indirectly promote angiogenesis at the fracture site, thereby facilitating fracture healing.

### YAP and β-catenin activities were increased after Piezo1 activation

**Figure 6:**
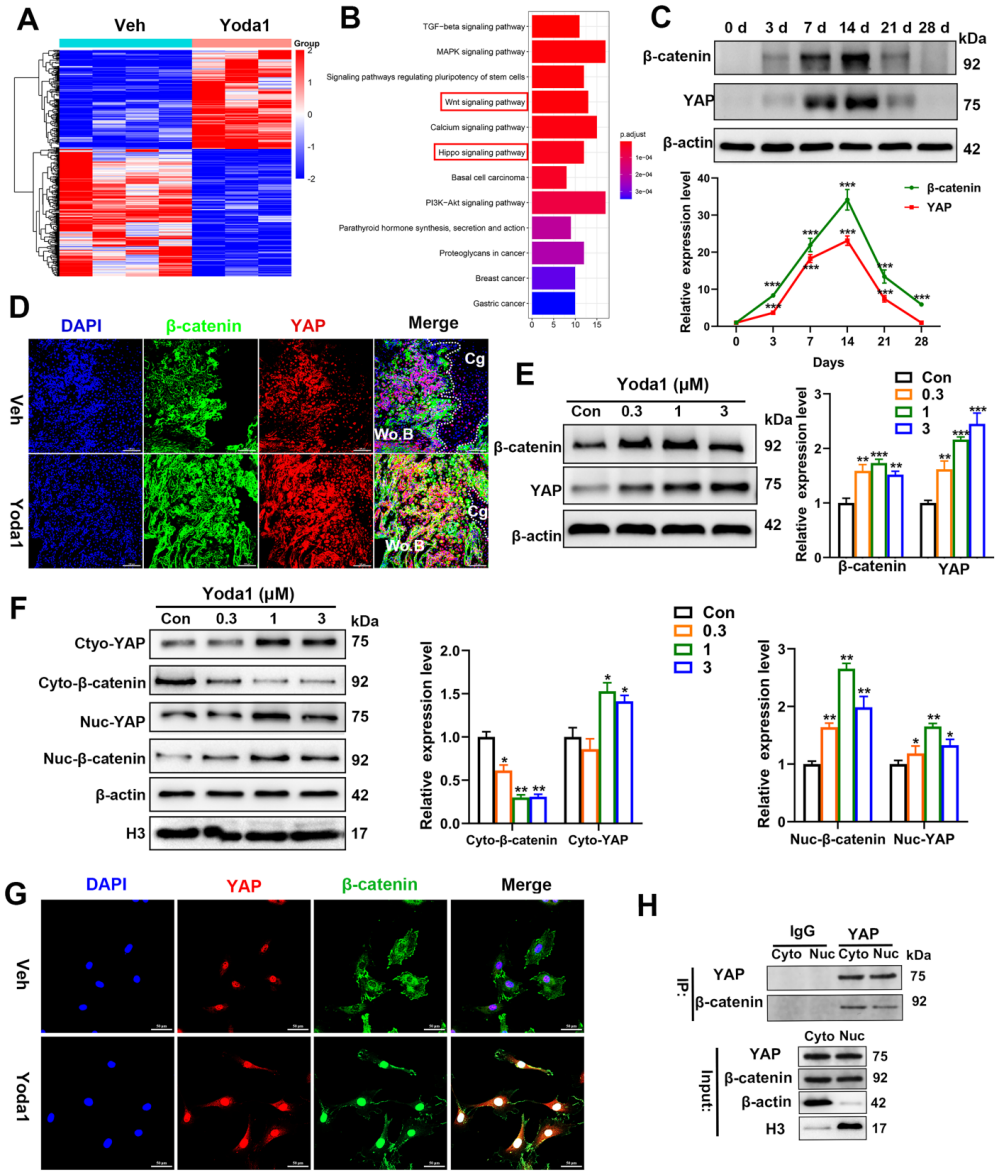
**YAP and β-catenin activities were increased after Piezo1 activation. (A)** Heatmap depicting expression profiling of the differentially expressed genes (DEGs) between the vehicle- and Yoda1-treated groups. **(B)** Kyoto encyclopedia of genes and genomes (KEGG) pathway analysis of DEGs. **(C)** YAP and β-catenin protein expression levels in fracture callus at 0, 3, 7, 14, 21 and 28 days post-fracture quantified by western blotting. Data are mean ± SD, n = 3 per group, ***p < 0.001. **(D)** Representative IF images of fracture callus at 10 days post-fracture, immunostained with β-catenin (green) and YAP (red) antibodies and counterstained with DAPI (blue). Dotted squares indicate areas magnified in woven bone. Scale bars = 20 µm. **(E)** Western blotting analysis of YAP, and β-catenin signaling-associated proteins in PSCs. Cells were stimulated with different concentrations of Yoda1 or vehicle for 24 h. Data are means ± SD of three independent experiments, **p < 0.01, ***p < 0.001. **(F)** Western blotting analysis of YAP and β-catenin proteins in the cytoplasm and nucleus of PSCs. Cells were stimulated with different concentrations of Yoda1 or vehicle for 24 h. Cyto, cytoplasm; Nuc, nucleus. β-actin and H3 are the loading controls. Data are means ± SD of three independent experiments, *p < 0.05, **p < 0.01. **(G)** Immunofluorescence staining of YAP and β-catenin in PSCs after treatment with Yoda1 (1 µM) or vehicle, as determined by confocal laser scanning microscopy. Scale bars = 50 µm. **(H)** Co-immunoprecipitation analysis. Nuclear and cytoplasmic PSC lysates were immunoprecipitated with anti-YAP or non-specific (ns) IgG after treatment with Yoda1 (1 µM) for 24 h. The resulting precipitates were subjected to immunoblotting analysis using the indicated antibodies. Input, ~50 µg lysate. Data are means ± SD of three independent experiments. Wo.B = woven bone; Cg = cartilage; Veh = vehicle.

In order to further understand the underlying mechanism of Piezo1 activation, we conducted RNA-Seq analysis of callus tissue from the vehicle-treated group and the Yoda1-treated group (Figure 6A). We identified 384 DEGs with 168 upregulated genes (including YAP, Ctnnb1, Bmp2 and Vegfa) and 216 downregulated genes in the Yoda1-treated group (Supplementary Figure S5A). Gene Ontology (GO) analysis revealed that these DEGs were highly relevant to the positive regulation of ossification, tissue development and osteoblastic differentiation (Figure S5B). Kyoto Encyclopedia of Genes and Genomes (KEGG) pathway analysis revealed that the DEGs were enriched in the Wnt and Hippo signaling pathways (Figure 6B).

The Wnt/β-catenin signaling pathway plays an important role during both osteogenesis and angiogenesis 43, 44. YAP is an essential downstream effector of the Hippo signaling pathway and regulates cell differentiation and bone formation 45, 46. Thus, the expression of key factors in these signaling pathways was investigated after Yoda1 treatment. Interestingly, the expression of YAP and β-catenin both increased during fracture healing, peaked by day 14 and decreased at day 21 and day 28 (Figure 6C). Moreover, Yoda1 treatment increased the expression of YAP and β-catenin in the woven bone formation area (Figure 6D). Moreover, treatment of PSCs with Yoda1 *in vitro* significantly increased total YAP and β-catenin expression (Figure 6E) and nuclear co-localization (Figure 6F, G) compared to that of vehicle. In addition, according to the Co-IP results, YAP interacted with β-catenin in the nucleus and cytoplasm (Figure 6H). Therefore, these results suggest that YAP may form a dimer with β-catenin that drives downstream gene expression of PSCs to promote osteogenesis and angiogenesis.

### Piezo1 promoted PSC-mediated differentiation and angiogenic effects through the YAP/β-catenin pathway

**Figure 7:**
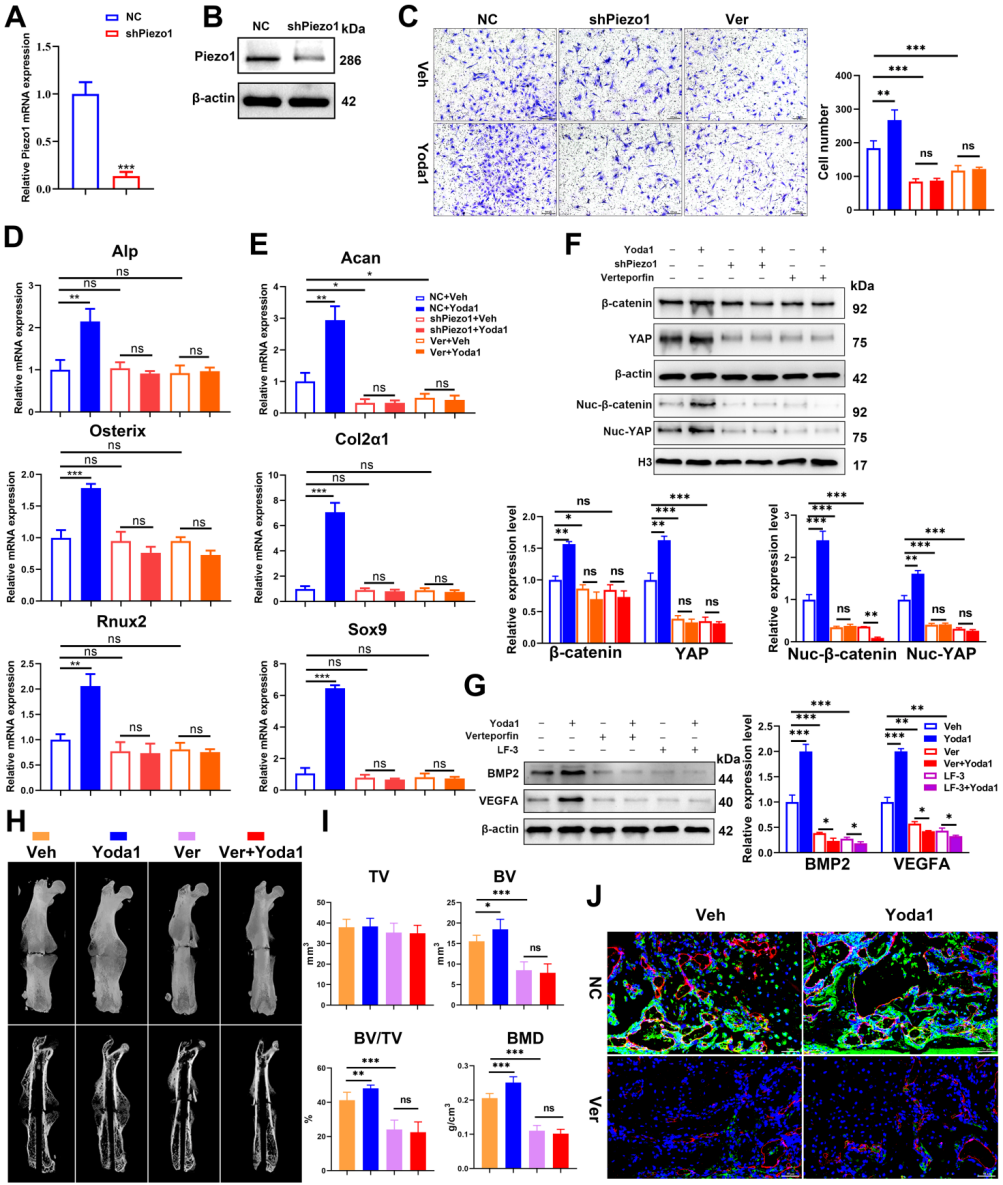
** YAP is an important mediator during Piezo1 activation. (A)** Relative expression of Piezo1 mRNA in PSCs after transfection with NC shRNA or Piezo1 shRNA for 72 h. Data are means ± SD of three independent experiments, ***p < 0.001. **(B)** Relative expression of Piezo1 protein in PSCs after transfection with NC shRNA or Piezo1 shRNA for 72 h. **(C)** Representative micrographs (left) and quantification (right) of the transwell migration assay in the indicated PSCs stimulated with Yoda1 for 24 h. Scale bars = 100 µm. Data are means ± SD of three independent experiments, **p < 0.01, ***p < 0.001*,* ns = no significant difference. **(D)** RT-PCR analysis of osteogenic marker gene expression in the indicated PSCs harvested after 10 days of culture in osteogenic medium with or without Yoda1 treatment. Data are means ± SD of three independent experiments, **p < 0.01, ***p < 0.001, ns = no significant difference. **(E)** RT-PCR analysis of chondrogenic marker gene expression in the indicated PSCs harvested after 10 days of culture in chondrogenic medium with or without Yoda1 treatment. Data are means ± SD of three independent experiments, *p < 0.05, **p < 0.01, ***p < 0.001. **(F)** Western blotting analysis of YAP and β-catenin signaling-associated proteins in PSCs. Cells were stimulated with or without Yoda1 (1 µM) for 24 h. Data are means ± SD of three independent experiments, *p < 0.05, **p < 0.01, ***p < 0.001. **(G)** Western blotting analysis of BMP2 and VEGFA proteins in the PSCs. Cells were stimulated with or without Yoda1 (1 µM) for 24 h. Data are means ± SD of three independent experiments, **p < 0.01, ***p < 0.001. **(H)** Representative three-dimensional reconstructions and coronal cross-sectional images of fracture calluses at 2 weeks post-fracture in the indicated mice. **(I)** Callus tissue volume (TV), bone volume (BV), bone volume fraction (BV/TV) and bone mineral density (BMD) of fracture calluses measured at 2 weeks post-fracture in the indicated mice. Data are means ± SD, n = 6 per group, *p < 0.05, **p < 0.01, ***p < 0.001, ns = no significant difference. **(J)** Representative IF images of fracture callus in the indicated mice at 14 days after fracture, immunostained with Emcn (red) and OCN (green) antibodies and counterstained with DAPI (blue). Scale bars = 50 µm. Veh = vehicle; Ver = verteporfin.

To further clarify the signaling pathway involved, Piezo1 shRNA and verteporfin, a YAP inhibitor, were used to block its signal transduction in PSCs which were subsequently treated with Yoda1. The expression of Piezo1 was significantly decreased by Piezo1 shRNA compared with nonspecific shRNA (Figure 7A, B) and downregulation of Piezo1 was found to suppress Yoda1-evoked Ca^2+^ entry into PSCs (Figure S6). In addition, migration of PSCs was similarly suppressed by Piezo1 knockdown and YAP inhibition, and the pro-migration effect of Yoda1 was also blocked (Figure 7C). These results indicate that migration of PSCs activated by Piezo1 activation is driven through YAP. Next, we examined the role of Piezo1 and YAP in PSC differentiation. The results showed that Piezo1 shRNA or YAP inhibitor did not significantly affect the expression of osteoblastic (Alp, Osterix and Runx2) or chondrogenic (Col2α1 and Sox9) markers. However, Yoda1-induced expression of osteoblastic and chondrogenic markers was significantly inhibited when Piezo1 expression was knocked down by Piezo1 shRNA or YAP inhibitor (Figure 7D-E). These results demonstrated that Piezo1 activates YAP expression and subsequently regulates the expression of osteogenic and chondrogenic genes. To verify the downstream pathway of YAP, we found that Yoda1-induced β-catenin expression and translocation into the nucleus were completely abolished by YAP inhibitor (Figure 7F), suggesting that Yoda1-induced β-catenin expression and nuclear location are YAP-dependent. To further determine the regulatory mechanism of BMP2 and VEGFA expression by YAP and β-catenin, we utilized the chemical compound verteporfin and LF-3, a β-catenin inhibitor, to block its downstream signaling. As shown in Figure 7G, Yoda1-induced BMP2 and VEGFA expression was effectively inhibited in PSCs. Taken together, these results reveal that Yoda1 activated the Piezo1/YAP/β-catenin signaling pathway of PSCs, which upregulated various osteogenic, chondrogenic and angiogenic factors, facilitating fracture healing.

In order to validate the conclusion *in vivo*, verteporfin was injected intraperitoneally into mice followed by Yoda1 (Figure 7H). μCT analysis showed that administration of verteporfin did not affect TV, but significantly reduced BV and BV/TV at 14 dpf. Importantly, Yoda1-induced accelerated fracture healing, with a higher BV and BV/TV, was completely abolished by verteporfin (Figure 7I). Consistent with the results of μCT, administration of verteporfin resulted in fewer OCN-positive cells and a reduced number of blood vessels in the callus region compared to the vehicle-treated group at 14 dpf (Figure 7J). However, chondrocyte differentiation did not appear to be significantly affected by YAP inhibition (Figure S7A-B). Administration of Yoda1 also failed to enhance formation of mineralized bone in the callus region of verteporfin-treated mice compared with the control group at day 14 (Figure 7I and Figure S7B), when the maximal amount of mineralized bone was detected. Additionally, mechanical testing showed that the peak load of fractured bones decreased by 53% at verteporfin-treated mice (Figure S7C). These data indicate that delivery of YAP inhibitors significantly blocks the fracture-healing effects of Piezo1 activation.

### Yoda1 injection mimicked the mechanical stimulation improvement of delayed fracture healing caused by mechanical unloading

**Figure 8:**
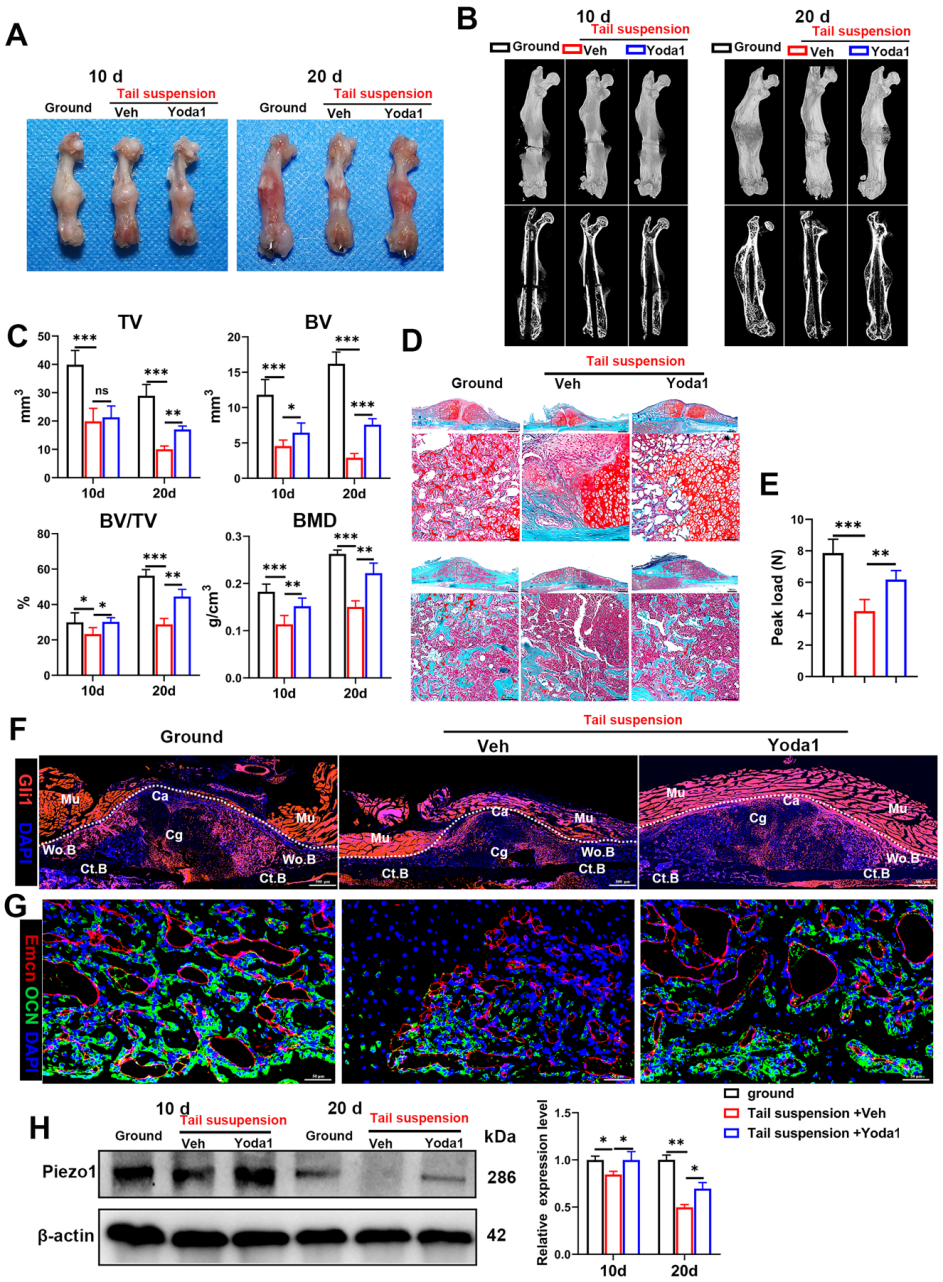
** Yoda1 treatment mitigated delayed fracture healing in tail-suspended mice. (A)** Representative images of fractured femora isolated from the indicated mice at 10 and 20 days post-fracture. Male mice were subjected to tail-suspension or ground control, as indicated. **(B)** Representative three-dimensional reconstructions and coronal cross-sectional images of fractured femora isolated from the indicated male mice at 10 and 20 days post-fracture. **(C)** Callus tissue volume (TV), bone volume (BV), bone volume fraction (BV/TV) and bone mineral density (BMD) of fracture calluses measured at 10 and 20 days post-fracture in the indicated mice. Data are means ± SD, n = 6 per group, *p < 0.05, **p < 0.01, ***p < 0.001, ns = no significant difference. **(D)** Representative Safranin O/Fast green staining images of fracture calluses at 10 and 20 days post-fracture. Scale bar = 500 µm (upper image); 100 µm (lower image). **(E)** Three-point bending test was performed on femora at 21 days post-fracture. Data are mean ± SD, n = 4 per group, **p < 0.01, ***p < 0.001. **(F)** Fluorescence images of femoral callus sections from the indicated Gli1/Tomato mice (n = 3 per group) at 10 days after fracture. Scale bar = 500 µm. **(G)** Representative IF images of fracture callus in indicated mice at 14 days after fracture, immunostained with Emcn (red) and OCN (green) antibodies and counterstained with DAPI (blue). Scale bars = 50 µm. **(H)** Piezo1 protein expression levels in fracture callus in the indicated mice at 10 and 20 days post-fracture, quantified by western blotting. Data are mean ± SD, n = 3 per group, *p < 0.05, **p < 0.01. Ct.B = cortical bone; Ca = callus; Cg = cartilage; Mu = muscle; Veh = vehicle; Wo.B = woven bone.

As the pathophysiology of fracture delayed union or nonunion is totally different from normal healing, we next explored whether activated Piezo1 had any role in delayed union caused by weightlessness or disuse 21. As shown in Figure 8A and B, tail suspension resulted in reduced total callus size at early (10 dpf) and late (20 dpf) time-points. Compared to the ground control group, BV, TV, BV/TV and BMD of the fracture callus were all reduced in the tail-suspension group, indicating delayed union. Strikingly, compared to the control group, administration of Yoda1 enhanced formation of mineralized bone, with higher BV, BV/TV and BMD in the callus of tail-suspension mice (Figure 8C). Moreover, the tail-suspended mice showed reduced fracture callus size. The fracture repair process was delayed with more cartilage remaining at 10 dpf, and with less mineralized bone formation at 20 dpf (Figure 8D). Mechanical testing of fractured bones at 20 days showed that the tail-suspended mice had 47% decreased peak load (Figure 8E). However, the delayed repair process was improved by Yoda1 treatment. In addition, in Gli1/Tomato mice, tail suspension resulted in far fewer Td+ cells in the callus, but Yoda1 treatment greatly rescued this effect (Figure 8F). Furthermore, compared to the control mice, the tail-suspended mice showed fewer OCN-positive cells and a reduced number of blood vessels in the callus region at 14 dpf, while Yoda1 treatment significantly rescued those effects (Figure 8G). What is even more interesting was that the Piezo1 protein expression level was significantly reduced in callus tissue at 20 days after tail suspension, and supplementation with Yoda1 reversed this decline in tail-suspension mice (Figure 8H). These findings demonstrated that mechanosensitive Piezo1 plays a dominant role in fracture healing by sensing mechanical stimuli, and that activation of Piezo1 by Yoda1 significantly reversed fracture delayed union by restoring the ability of PSCs to undergo osteogenic differentiation at the fracture site.

## Discussion

In this study, we found that the Piezo1 channel acts as a regulator of fracture healing by sensing mechanical loading. We first provided deep insights into the cellular subpopulations during the fracture-healing process by scRNA-seq, and clarified expression patterns of Piezo proteins (Piezo1 and Piezo2) in the callus tissue. Intriguingly, we found that Piezo1 was enriched in PSCs and their derived osteoblastic lineage cells and chondrocytes, and was upregulated during the fracture-healing process, indicating a strong association of Piezo1 and fracture healing. Further downregulation of Piezo1 in fracture callus impaired the healing process, while chemical activation accelerated fracture healing by promoting bone regeneration. These findings are generally compatible with biomechanical stimulation during osteogenesis 10. Next, mechanistic study suggested that Piezo1 regulated YAP-β-catenin signaling, which upregulated expression of BMP2 and VEGFA in PSCs, two most effective molecules for osteogenesis and angiogenesis. Finally, we demonstrated that chemical activation of the Piezo1 channel mimics mechanical stimuli, improving fracture healing in a mouse model of delayed union generated by tail suspension. Taken together, these results indicate that Piezo1, a mechanically activated ion channel, is a potential therapeutic target for fracture healing and delayed union, especially when caused by weightlessness or disuse.

Multiple cells in the fracture site including PSCs, osteoblastic lineage cells, inflammatory cells, endothelial cells and so on are all involved in the fracture healing process, so accurately identifying the specific targeted cell of action first is of great importance. By scRNA-seq, we divided callus cells into 15 clusters, and found that PSCs and their derived osteoblastic lineage cells and chondrocytes constituted a large portion of the callus cells. Moreover, Piezo1 was highly enriched in this cluster, indicating that PSCs and their derived cells are the main responding cells of the Piezo1 pathway. Even though numerous studies have identified the role of Piezo1 in bone formation, they mainly focused on bone marrow MSCs, which differ greatly from PSCs 47. Consequently, our single-cell resolution data followed by a series of functional experiments are the first to highlight the role of Piezo1 in PSC-mediated fracture healing and in delayed union.

Piezo channels have crucial roles in various mechanotransduction processes. Whereas Piezo2 is mainly expressed in dorsal root ganglia neurons regulating mechanical nociception 48, Piezo1 is expressed in non-neuronal tissues with a broader physiological role 14, 16, 19. Similarly, in this study, our scRNA-seq data revealed that Piezo1 was highly expressed in cells involved in bone repair relative to Piezo2. Knockdown of Piezo1 resulted in impaired PSC function and thus abnormal fracture healing. Moreover, our results showed that activation of Piezo1 using Yoda1 enhanced chondrogenesis in cartilage callus formation at 14 dpf and accelerated cartilage turnover and mineralized bone formation at 28 dpf. Previous studies showed that mechanical stimuli induce intramembranous and endochondral ossification, and promote fracture healing 49. In addition, biophysical stimuli such as mechanical strain, fluid shear stress (FSS), microgravity and vibration regulate non-coding RNA expression, thereby regulating the process of osteogenic differentiation 50, 51. Piezo1 as a major mechanosensor is necessary for mechanical stimulation to promote bone formation 21, 50. Therefore, Piezo1 can act as a promising therapeutic target to promote fracture healing.

Previous studies have shown that the Piezo1 channel regulates the migration and osteoblastic differentiation of BMSCs 52, 53. However, the viability of PSCs is totally different from that of BMSCs 47 and they are the major cell sources of osteoblast lineage cells and chondrocytes in the callus for fracture healing 4, 31, 54; however the effects of Piezo1 on cell fate determination of PSCs are not characterized yet. Our data suggest that activation of Piezo1 promotes migration and osteoblastic differentiation of PSCs. Interestingly, the chondrogenic differentiation of PSCs was also enhanced following Piezo1 activation. Consistent with effects in myogenesis, the Piezo1 channel promotes PSC migration and osteogenic differentiation rather than proliferation during bone regeneration 55. In addition, activation of Piezo1 in periosteal CD68^+^F4/80^+^ macrophages also contributed greatly to periosteal bone formation by secretion and activation of TGF-β1 56. Our study showed that activation of Piezo1 in PSCs resulted in enhanced VEGF secretion and thus indirectly promoted HUVEC migration and tube formation *in vitro,* enhancing type H vessel formation in fracture callus. A previous study has shown that Piezo1 directly regulates vessel function 16, and our scRNA-seq data also revealed moderate expression of Piezo1 in endothelial cells. Therefore, we conclude that Piezo1 activation by Yoda1 could promote vessel growth in the callus via both direct and indirect mediation by PSCs. In conclusion, Piezo1 activation enhanced the chondrogenesis and osteogenesis of PSCs, and also increased angiogenic effects by upregulating VEGFA secretion from PSCs. All these effects contributed to accelerated cartilage callus formation and later cartilage-to-bone transformation.

By mechanotransduction systems, cells convert mechanical stimuli into biochemical signals so as to control multiple aspects of cell behavior, including differentiation and growth as well as cancer malignant progression 57, 58. YAP are important transcriptional factors that are widely known as mechanosensors and mechanotransducers in various cell types 58, 59. Additionally, the Wnt effector β-catenin is also regulated by mechanical cues 60. In our study, YAP and β-catenin were activated synchronously in PSCs during fracture repair in mice. Yoda1 treatment increased the expression and nuclear localization of YAP and β-catenin both *in vitro* and *in vivo*. The overlap between YAP and β-catenin regulation in fracture repair suggest that these factors might have a potential crosstalk, but the mechanism involved in fracture healing has not yet been investigated. However, recent studies have revealed that YAP-β-catenin is an important pathway for osteogenesis, enterocyte regeneration, and intervertebral disc degeneration 61-63. In addition, YAP has been shown to interact with β-catenin and form a complex in the nucleus to regulate downstream genes 62, 64, 65. *In vitro*, knockdown of Piezo1 in PSCs abolished the increase in YAP and β-catenin expression levels and nuclear accumulation following Yoda1 stimulation. Thus, activation of YAP and β-catenin may be a master regulatory mechanism of Piezo1-mediated fracture healing.

YAP has recently emerged as a critical mediator of bone development and repair 66. However, conflicting reports exist regarding the function of YAP in fracture healing. Deng et al. reported that YAP governed the initiation of fracture healing by inhibition of cartilage callus formation 45, whereas Kegelman et al. found that YAP promoted the expansion and differentiation of periosteal osteoblast precursors, but had no effect on cartilage formation during fracture repair 46. Consistent with Kegelman et al., our results support the theory that YAP inhibition mainly impairs intramembranous bone formation and angiogenesis but has no effect on cartilage callus formation. Furthermore, previous studies showed that Piezo1 deficiency did not impact osteoblast differentiation 21, and that deletion of YAP from osteoprogenitor cells increased osteoblast differentiation *in vitro* 67. Our work here demonstrates that neither Piezo1 knockdown nor YAP inhibition affect PSC differentiation but instead inhibit migration. However, Yoda1-mediated promotional effects were completely blocked by Piezo1 knockdown and YAP inhibition *in vitro*. Importantly, inhibition of YAP activity *in vivo* was able to block the excitatory effect of Piezo1 activation in fracture healing. Therefore, YAP serves as a downstream effector of the Piezo1 channel in fracture healing. The Wnt/β-catenin signaling pathway is well known to play major roles in bone development and fracture repair. Several studies have suggested that β-catenin is linked to YAP activity 61, 62. In the present study, we found that inhibition of YAP activity by verteporfin effectively attenuated β-catenin expression and nuclear translocation by Piezo1 activation. Thus, YAP is a key upstream molecule required for the osteogenic activity of β-catenin in bone regeneration 63.

VEGFA and BMP2 have been shown to be critical to bone formation during fracture repair 42, 68, being involved in angiogenesis and osteogenesis and mutually regulating each other's biological function 69. In this study, we found that both BMP2 and VEGFA expression in PSCs were increased following Yoda1 treatment *in vitro* and *in vivo*. Interestingly, inhibition of YAP activity by verteporfin blocked increased BMP2 and VEGFA levels, and the same results were observed following inhibition of β-catenin activity with LF-3. Consistently, previous studies have reported that BMP2 and VEGFA are both regulated by β-catenin signaling 70-72. These observations suggest that activation of the Piezo1 channel by Yoda1 might stimulate BMP2 and VEGFA expression through a YAP-β-catenin signaling pathway in PSCs.

Piezo1 is an essential mechanosensor in bone homeostasis and is highly sensitive to mechanical loading. Moreover, SNPs of the Piezo1 locus are associated with osteoporosis and increased fracture risk in humans 73. Mechanical loading increases the expression of Piezo1 while mechanical unloading reduces its expression. Therefore, a positive feedback loop exists between the mechanosensitive Piezo1 and the mechanical stimulus 24, 74. Similarly, in our study, tail suspension impaired callus formation and ossification. Piezo1 activation mimicked the effects of mechanical stimulation to improve fracture healing and maintain Piezo1 expression levels in the bone callus in tail-suspended mice. Therefore, activating Piezo1 may represent a promising potential therapeutic target for preventing or treating mechanical unloading-induced fracture delayed union or nonunion.

In summary, activation of Piezo1 in PSCs accelerated fracture healing. Further studies using inducible Piezo1 conditional-knockout mice specifically in PSCs, such as Gli1-CreER Piezo1^flox/flox^ and LeptinR-CreER Piezo1^flox/flox^ mice are required to further elucidate spatiotemporal target cells and actions of this pathway and its effects on fracture healing. Nevertheless, our study supports the idea that therapeutic agents targeting Piezo1 could be considered as new targets for the treatment of delayed or nonunion, especially when caused by abnormal biomechanics.

## Supplementary Material

Supplementary figures and tables.

## Abbreviations

CCK-8: cell counting kit-8
BMP2: bone morphogenetic protein 2
BMSCs: bone marrow mesenchymal stem cells
BMD: bone mineral density
BSA: bovine serum albumin
BV: bone volume
Co-IP: co-immunoprecipitation
DAPI: 4',6-diamidino-2-phenylindole
DEGs: differentially expressed genes
dpf: days post fracture
FBS: fetal bovine serum
GO: gene ontology
HUVECs: human umbilical vein endothelial cells
IHC: immunohistochemistry
KEGG: Kyoto encyclopedia of genes and genomes
Lv: lentivirus
MFI: mean fluorescence intensity
NC: negative control
OCN: osteocalcin
PFA: paraformaldehyde
PSCs: periosteal stem cells
RT-PCR: real-time polymerase chain reaction
scRNA-seq: single cell RNA sequencing
SD: standard deviation
TV: total volume
μCT: micro-computed tomography
UMAP: uniform manifold approximation and projection
VEGFA: vascular endothelial growth factor A
YAP: yes-associated protein
