# Supplementary material for: Mechanosensitive Piezo1 is crucial for periosteal stem cell-mediated fracture healing
Source: Int J Biol Sci. 2022 Jun 13;18(10):3961–80. doi: 10.7150/ijbs.71390 (PMC9274506; doi:10.7150/ijbs.71390)
Supplement: Supplementary file 1 — Supplementary figures and tables. [file ijbsv18p3961s1.pdf]

Figure S1

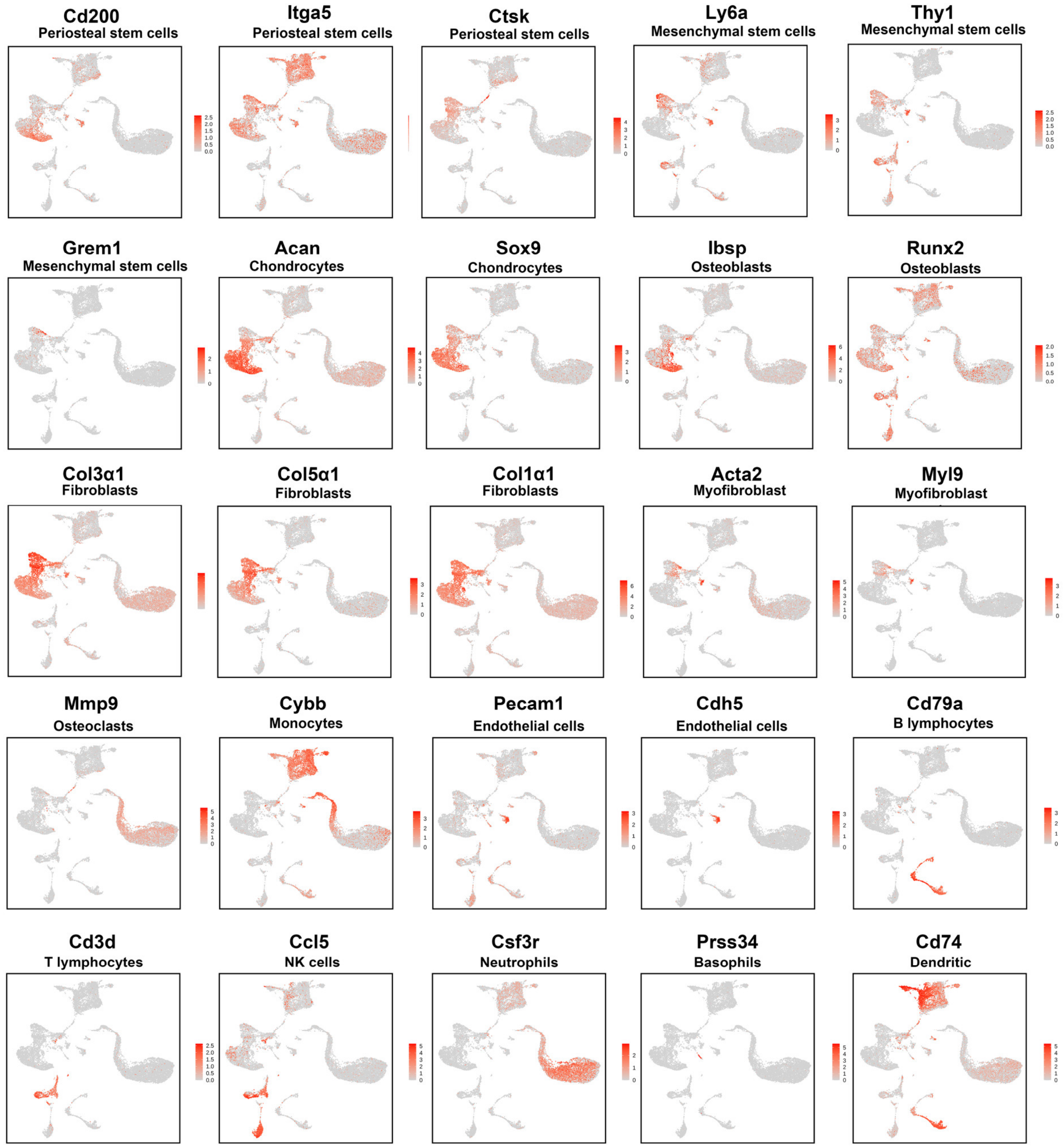

Figure S1. Expression of marker genes for cell populations highlighted on UMAP.

Figure S2

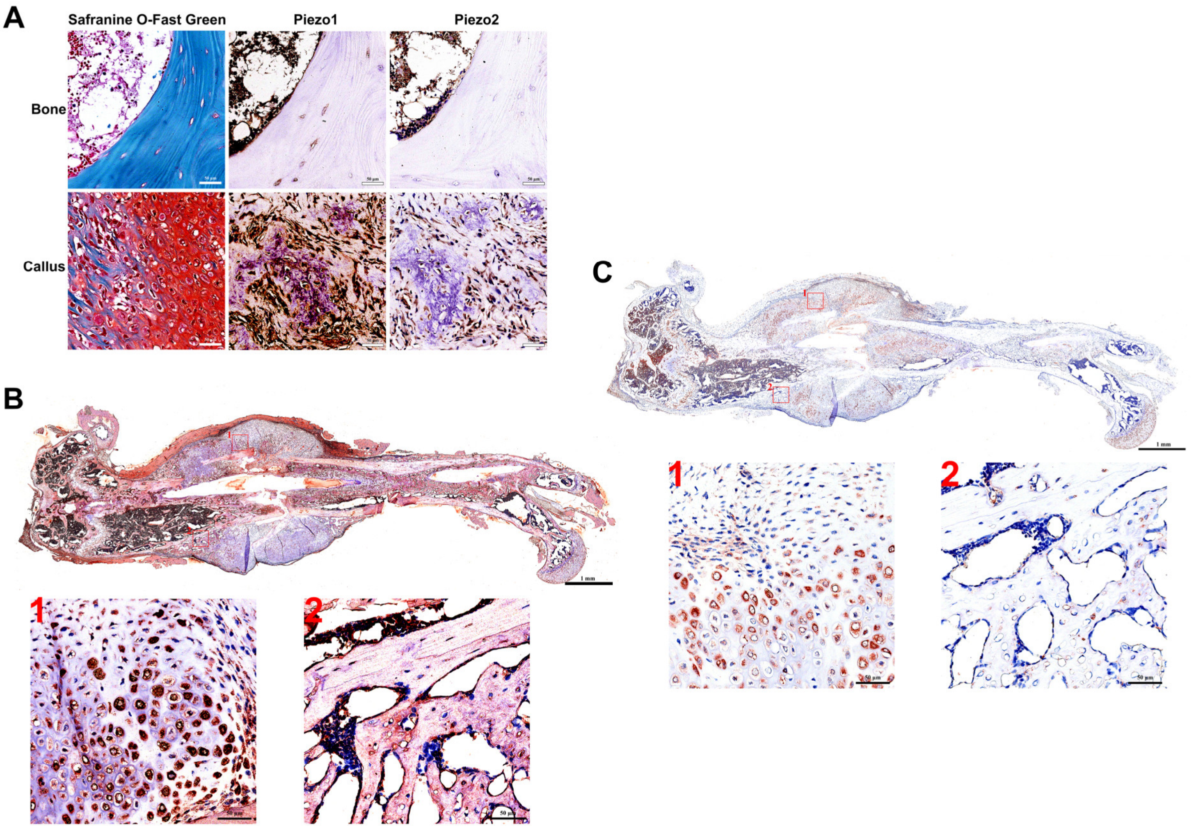

Figure S2. Piezo protein expression pattern in human and mouse callus tissue. (A) Safranin O/Fast green (1st column) and IHC staining of Piezo1 (2nd column) and Piezo2 (3rd column) in human fracture callus and normal bone sections. Scale bars = 50 μm. (B) IHC staining of Piezo1 in mouse fracture callus sections at 10 days post-fracture. Scale bar = 1 mm (upper image); 50 μm (lower image). (C) IHC staining of Piezo2 in mouse fracture callus sections at 10 days post-fracture. Scale bar = 1 mm (upper image); 50 μm (lower image).

Figure S3

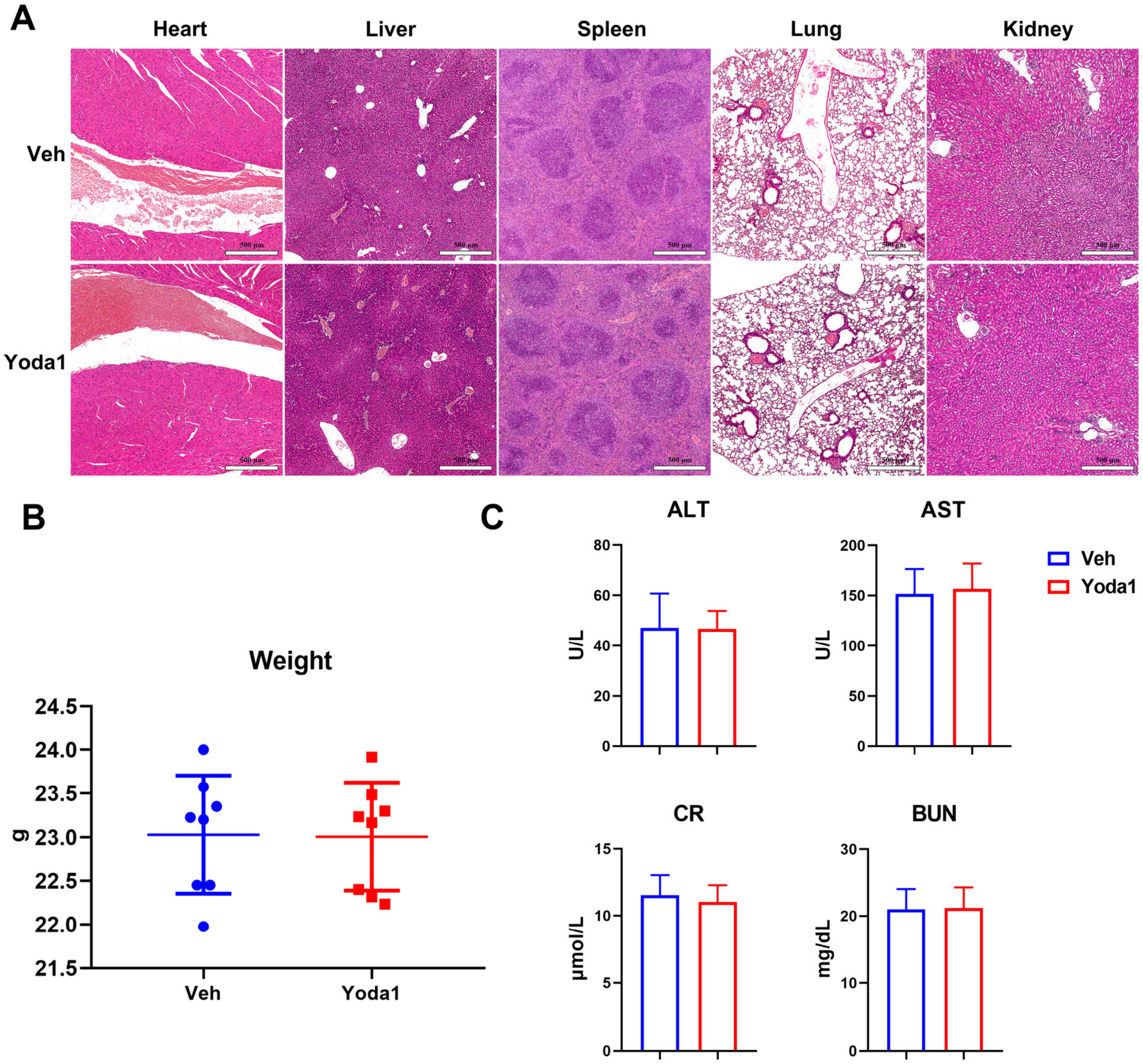

Figure S3. Physiological function analysis after Yoda1 administration in mice. (A) Representative hematoxylin-eosin staining images of heart, liver, spleen, lung and kidney isolated from mice after intraperitoneal injection of vehicle or Yoda1 for 4 weeks. (B) Body weight was measured after 4 weeks of intraperitoneal injection. Data are means ± SD, n = 6 per group. (C) Hematology tests of liver and kidney functions of the mice following intraperitoneal injection of vehicle or Yoda1 for 4 weeks. Data are means ± SD, n = 6 per group. *ALT = alanine transaminase; AST = aspartate transaminase; BUN = blood urea nitrogen; CR = creatinine; Veh = vehicle.*

Figure S4

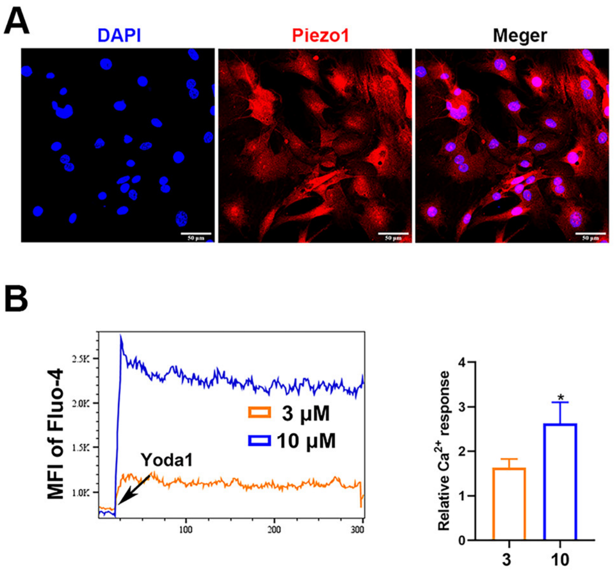

Figure S4. The expression of Piezo1 in PSCs and its effect on Yoda1 -induced Ca^{2+}response. (A) Cellular localizations of Piezo1 (red) in PSCs determined by immunostaining. Scale bars = 50 μm. (B) Calcium influx of PSCs stimulated by 3 μM or 10 μM Yoda1. Average trace of all cells indicating how Fluo-4 fluorescence changed over time (left). Scatter plot of baseline and Yoda1-induced Fluo-4 amplitude changes in PSCs (right). Data are means ± SD of three independent experiments, *$p < 0.05$. *MFI = mean fluorescence intensity*.

Figure S5.

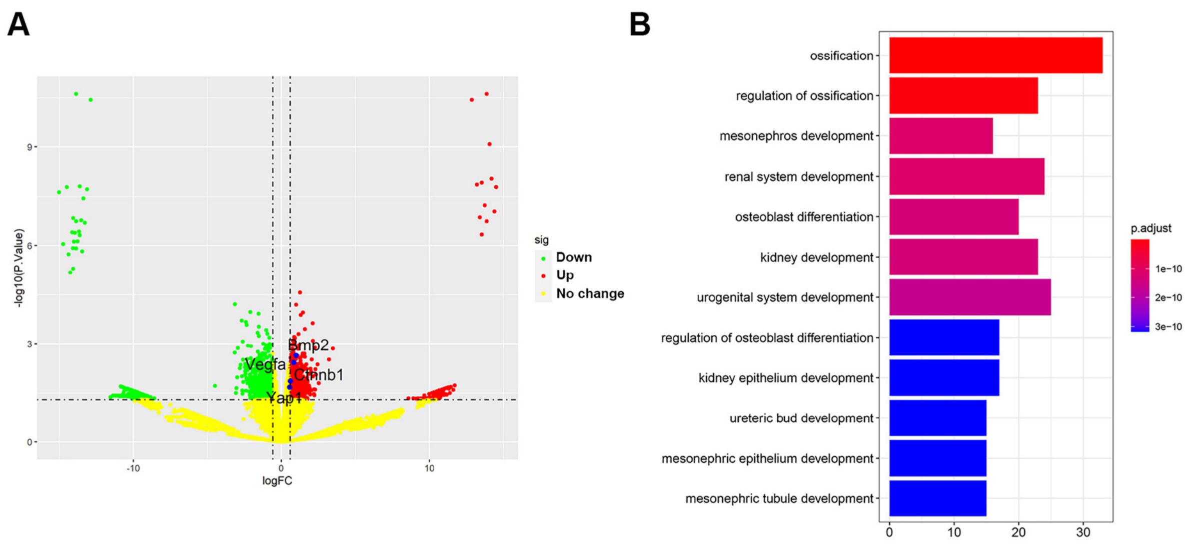

Figure S5. Quantitative transcriptome analysis of fracture callus between vehicle- and Yoda1-treated groups. (A) Volcano plot showing the differentially expressed genes (DEGs) comparing the Yoda1-treated group to the vehicle-treated group. (B) Gene ontology (GO) enrichment analysis of DEGs. *Veh = vehicle.*

Figure S6

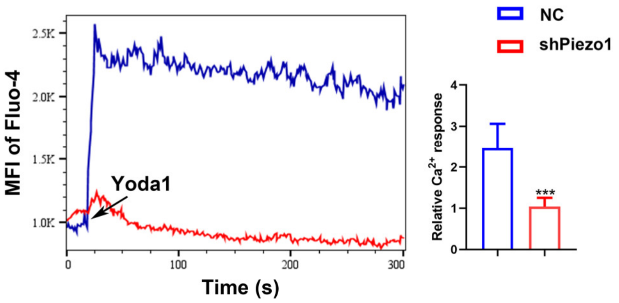

Figure S6. Effects of Piezo1 knockdown on Yoda1- induced Ca^{2+} response in PSCs. Calcium influx of PSCs stimulated by Yoda1 (10 μM). Cells were transfected with NC shRNA or Piezo1 shRNA for 72 h (left). Average trace of all cells indicated Fluo-4 fluorescence changed over time. Scatter plot of baseline and Yoda1-induced Fluo-4 amplitude changes in PSCs (right). Data are means ± SD of three independent experiments, ***$p < 0.001$. *MFI = mean fluorescence intensity*.

Figure S7

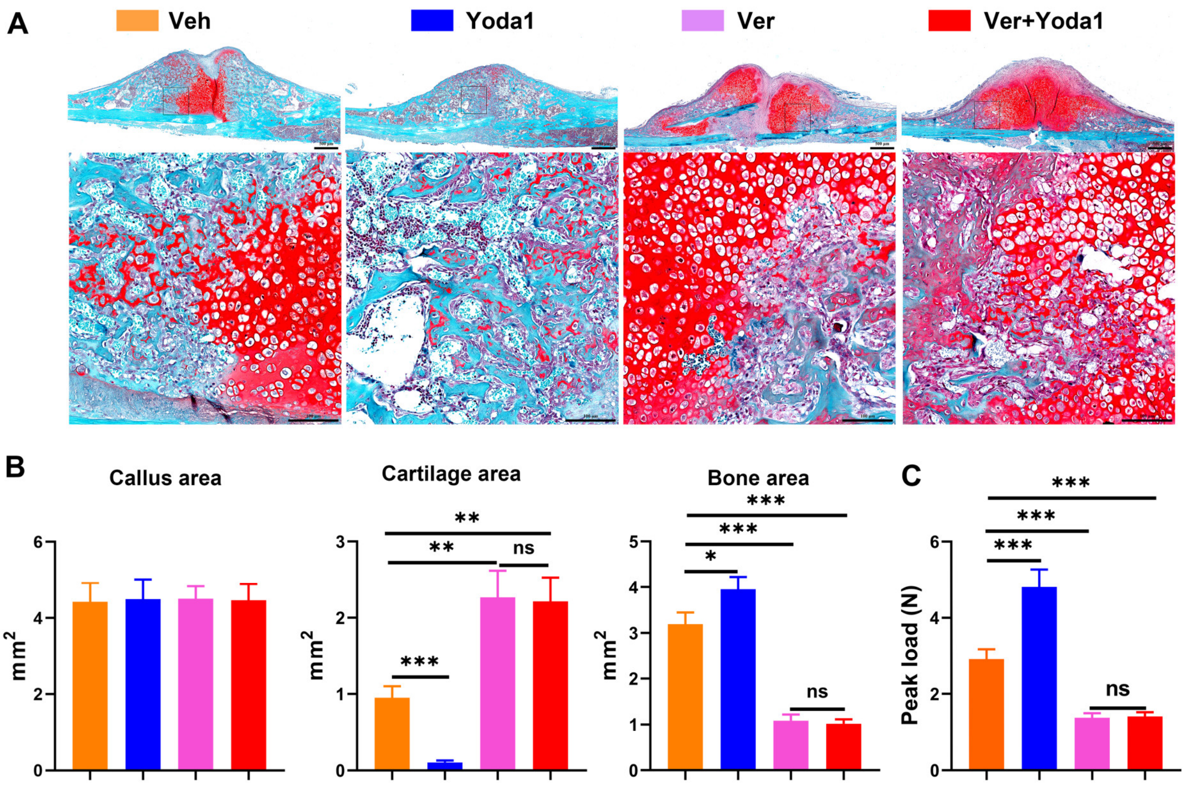

Figure S7. Effects of YAP inhibitors on fracture repair in mice. (A) Representative Safranin O/Fast green staining images of fracture calluses in the indicated mice at 14 days post-fracture. Scale bar = 500 μm (upper image); 100 μm (lower image). (B) Callus area, cartilage area, and bone area of the fracture callus measured in the indicated mice at 14 days post-fracture. Data are mean ± SD, n = 4 per group, *$p < 0.05$, **$p < 0.01$, ***$p < 0.001$. (C) Three-point bending test was performed on femora at 14 days post-fracture. Data are mean ± SD, n = 4 per group, ***$p < 0.001$. *Veh = vehicle; Ver = verteporfin.*

Table S1. Donors' information involved in this study

Case NO.	Age (years)	Gender	Diagnosis	Injury time (days)
P1	49	Male	Pelvic fracture	13
P2	16	Male	Bilateral Pilon fractures	42
P3	44	Male	Comminuted fracture of left femur and tibia	21
P4	51	Female	Fracture of right clavicle	17
P5	65	Female	Pelvic fracture	12

Table S2: Human and mouse real-time PCR primer sequences used in this study

Gene	Forward primer（5’ to 3’）	Reverse primer（5’ to 3’）
PIEZO1	AGATCTCGCACTCCAT	CTCCTTCTCACGAGTCC
PIEZO2	CACCTGGCTACAACTGCTCA	CCCGATGTCAGGTACAAACA
GAPDH	CAGGAGGCATTGCTGATGAT	GAAGGCTGGGGCTCATTT
Piezo1	GCTTGCTAGAACTTCACG	GTACTCATGCGGGTTG
Alpl	ATCTTTGGTCTGGCTCCCATG	TTTCCCGTTCACCGTCCAC
Runx2	ACGAAAAATTAACGCCAGTCG	TCGGTCTGACGACGCTAAAG
Osterix	AGAGGTTCACTCGCTCTGACGA	TTGCTCAAGTGGTCGCTTCTG
Col2α1	GCTCCCAGAACATCACCTACCA	TCATTGGAGCCCTGGATG
Acan	CCTTGTCACCATAGCAACCT	CTACAGAACAGCGCCATCA
Sox9	AAGGGCTACGACTGGACG	ATGCGGGTACTGGTCTGC
Gapdh	GGTTGTCTCCTGCGACTTCA	TGGTCCAGGGTTTCTTACTCC
